# Functional Analysis of Autoantibody Signatures in Rheumatoid Arthritis

**DOI:** 10.3390/molecules27041452

**Published:** 2022-02-21

**Authors:** Lisa Milchram, Anita Fischer, Jasmin Huber, Regina Soldo, Daniela Sieghart, Klemens Vierlinger, Stephan Blüml, Günter Steiner, Andreas Weinhäusel

**Affiliations:** 1 Center for Health and Bioresources, Molecular Diagnostics, AIT Austrian Institute of Technology GmbH, Giefinggasse 4, 1210 Vienna, Austria; lisa.milchram@ait.ac.at (L.M.); jasmin.huber@ait.ac.at (J.H.); regina.soldo@ait.ac.at (R.S.); klemens.vierlinger@ait.ac.at (K.V.); 2 Department of Internal Medicine III, Division of Rheumatology, Medical University of Vienna, Währinger Gürtel 18–20, 1090 Vienna, Austria; anita.fischer@meduniwien.ac.at (A.F.); daniela.sieghart@meduniwien.ac.at (D.S.); stephan.blueml@meduniwien.ac.at (S.B.); guenter.steiner@meduniwien.ac.at (G.S.); 3 Ludwig Boltzmann Institute for Arthritis and Rehabilitation, Medical University of Vienna, Währinger Gürtel 18–20, 1090 Vienna, Austria

**Keywords:** rheumatoid arthritis, autoantibodies, seroreactivity, disease activity, rat model, pathway analysis

## Abstract

For the identification of antigenic protein biomarkers for rheumatoid arthritis (RA), we conducted IgG profiling on high density protein microarrays. Plasma IgG of 96 human samples (healthy controls, osteoarthritis, seropositive and seronegative RA, *n* = 24 each) and time-series plasma of a pristane-induced arthritis (PIA) rat model (*n* = 24 total) were probed on AIT’s 16k protein microarray. To investigate the analogy of underlying disease pathways, differential reactivity analysis was conducted. A total of *n* = 602 differentially reactive antigens (DIRAGs) at a significance cutoff of *p* < 0.05 were identified between seropositive and seronegative RA for the human samples. Correlation with the clinical disease activity index revealed an inverse correlation of antibodies against self-proteins found in pathways relevant for antigen presentation and immune regulation. The PIA model showed *n* = 1291 significant DIRAGs within acute disease. Significant DIRAGs for (I) seropositive, (II) seronegative and (III) PIA were subjected to the Reactome pathway browser which also revealed pathways relevant for antigen presentation and immune regulation; of these, seven overlapping pathways had high significance. We therefore conclude that the PIA model reflects the biological similarities of the disease pathogenesis. Our data show that protein array analysis can elucidate biological differences and pathways relevant in disease as well be a useful additional layer of omics information.

## 1. Introduction

**Figure 1:**
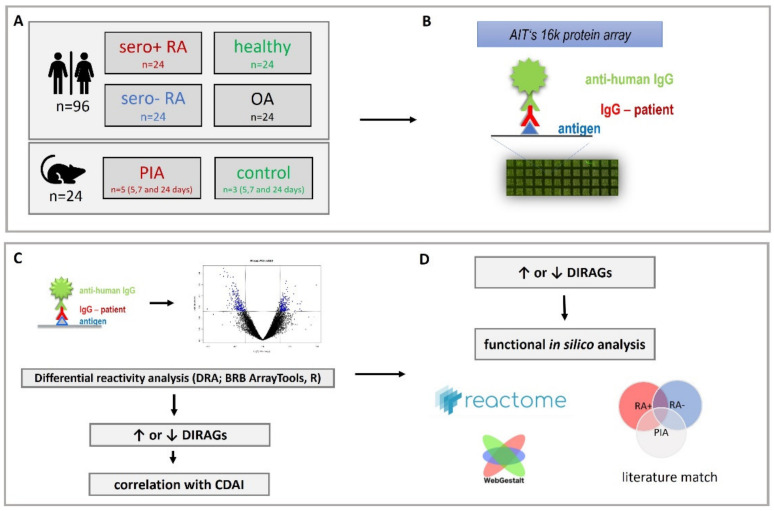
Study design. (**A**) sample cohort: 96 samples from RF- and CCP-positive (sero+), RF- and CCP- negative (sero-) RA, osteoarthritis and healthy human individuals and 24 samples from time-course pristane induced arthritis (PIA) and control animals were investigated. (**B**) IgG isolated from plasma was probed on AIT’s 16k microarray. (**C**) Data obtained from microarray scans was subjected to differential reactivity analysis (DRA) and correlation analysis with clinical disease activity index (CDAI) using BRB ArrayTools and RStudio elucidating differentially reactive antigens (DIRAGs) which were subsequently (**D**) in silico analyzed for dysregulated pathways.

Rheumatoid arthritis (RA) is a systemic autoimmune disease characterized by the presence of auto-reactive B- and T-cells, autoantibodies and increased cytokine release which all together lead to chronic joint inflammation. In untreated RA, fibroblasts and osteoclasts are activated triggering cartilage degradation and bone destruction [1]. The diagnosis relies on a combination of clinical serological and radiographic assessments accompanied by the EULAR classification criteria. The serological diagnosis is based on the presence of rheumatoid factor (RF) and anti-citrullinated protein/peptide antibodies (ACPAs) [2]. ACPAs and RF enable the differentiation of two serological groups of RA (seropositive and seronegative) and were shown to be of prognostic value for disease progression [3,4]. However, the pathological role of auto-antibodies is hardly understood, although extensive research is ongoing [5]. In the search for improved and novel therapeutics for RA, animal models play a major role in research and development. In a recent review, Meehan et al. described the currently available collection of preclinical models [6]. Besides the known advantages and limitations of these models, currently no comparative analysis of auto-antibody signatures for RA and its respective animal models can be found within the literature. Among these models pristane-induced arthritis (PIA) is of particular interest, because arthritogenic autoimmunity is induced in rats by the application of the non-immunogenic mineral oil pristane (2,6,10,14-Tetramethylpentadecane). PIA shows many features which are similar to human RA, such as chronic synovitis, cartilage degradation, bone erosions and the presence of RF [7]. Therefore, we conducted IgG profiling of human and rodent plasma on high density protein microarrays and subjected higher reactive differentially reactive antigens (DIRAGs) to pathway analysis aiming to elucidate the underlying processes (Figure 1).

## 2. Results

To elucidate and investigate auto-antibody signatures of RF- and CCP-positive and -negative rheumatoid arthritis and the PIA rodent model described by Tuncel et al., IgG probing was conducted on high density protein microarrays. IgG was isolated from treatment naive plasma of human individuals and rat sera and probed on AIT’s 16k protein microarray (comprising *n* = 7390 proteins recombinantly expressed in 15,417 cDNA *E.coli* clones). Data extracted from microarray images were analysed for differentially reactive antigens (DIRAGs) between seropositive and seronegative RA and samples of the PIA rat model upon the disease onset period. In addition, antibody reactivities in the human RA samples were correlated with the clinical disease activity index (CDAI). Lists comprising DIRAGs were further investigated in silico with the Reactome pathway browser and the WebGestalt analysis toolkit to investigate the underlying disease pathways (methodological details are given in the Methods Section 4). DIRAGs of suspected biological relevance (included in the top pathways) were evaluated for previously described roles as auto-antibodies or involvement in auto-immune diseases.

### 2.1. Antibody Isolation

The concentration of isolated IgG was determined via duplicate A280 measurements and averaged. Human IgG concentration ranged from 0.809–2.918 mg/mL (mean: 1.403 ± 0.362 mg/mL (plasma concentration: 10.240 ± 2.641 mg/mL)). Student’s *t*-test (*p* < 0.05) showed no significant differences between the concentrations of the groups. Mean rat IgG concentration ranged from 0.423–0.727 mg/mL (mean: 0.529 ± 0.063 mg/mL [plasma concentration: 3.861 ± 0.447 mg/mL]), without significant differences between the control and PIA group (Student’s *t*-test, *p* < 0.05). IgG integrity was determined via observed molecular weight determined within SDS-PAGE, a sharp band at 150 kDa was probative for structural protein integrity. An SDS-Page image of the purified IgG from human samples is given in Supplementary Materials Figure S1.

### 2.2. Differential Reactivity Analysis

**Figure 2:**
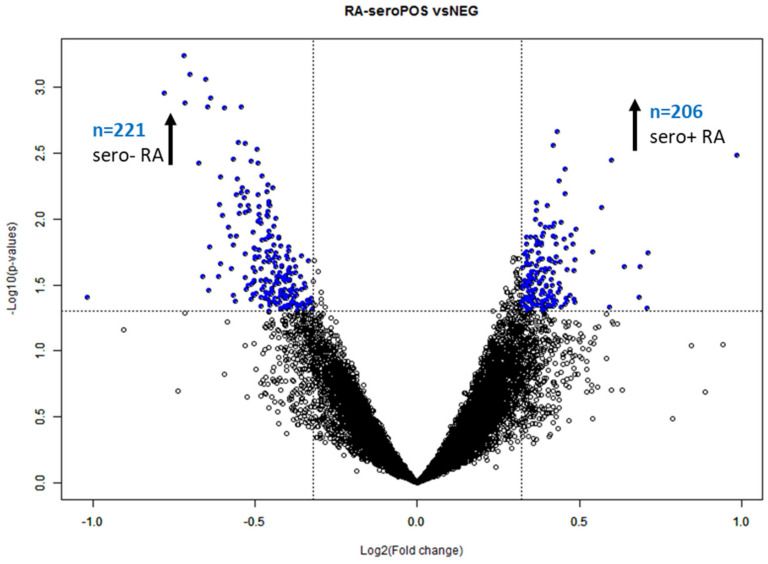
Volcano plot of seropositive versus seronegative RA. The unblocked class comparison elucidated *n* = 382 significant (*p* < 0.05) DIRAGs with a fold-change > 1.25 (−0.3219 and 0.3219 on the log2 scale, indicated as dashed line in the plot above). DIRAGs above the significance thresholds are indicated in blue (BRB ArrayTools [8] output per default). The sign of the fold-change is assigned in alphabetical order; hence, proteins higher reactive in seropositive RA are located on the left side of the plot.

Class comparison analysis (*p* < 0.05) was applied to the human IgG profiles for the groups seropositive versus seronegative RA to elucidate differentially reactive antigens (DIRAGs). Out of *n* = 15,032 features passing the filtering criteria, *n* = 382 proteins were found to be significantly differentially reactive (*p* < 0.05) with a fold-change of > 1.25 (Figure 2). Class comparison analysis was repeated with respect to the observed clusters with the assigned cluster as a blocking variable. Blocked analysis revealed *n* = 602 significant differential reactive features, *n* = 206 higher reactive DIRAGs in seropositive RA versus seronegative RA with unambiguous gene symbols used in pathway analysis. In seronegative RA, *n* = 221 higher reactive DIRAGs were used for Reactome analysis.

**Figure 3:**
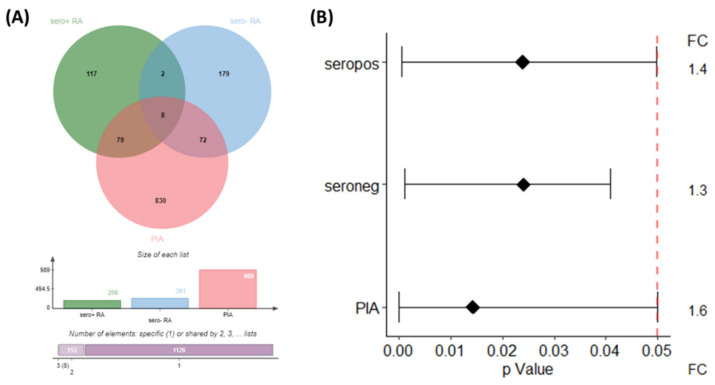
Intersection of significant higher reactive DIRAGs as Venn Diagram (**A**) and their respective *p*-values and the average fold-change as a forest plot (**B**). Results of the blocked analysis of the comparison seropositive versus seronegative RA was used, and PIA 7 vs. 24 days after correction for DIRAGs higher reactive in PBS animals. VennDiagram created with JVenn [9].

Class comparison (*p* < 0.05) applied to the rat samples between 7 and 24 days after arthritis induction with pristane revealed *n* = 1766 (PIA) and *n* = 1352 (controls) significant DIRAGs with a fold-change > 1.35 (*p* < 0.05). After correction for significant DIRAGs higher reactive in the control animals, class comparison (*p* < 0.05) for PIA revealed *n* = 1291 significant DIRAGs with a fold-change > 1.35. *n* = 988 DIRAGs remained after gene symbol cleansing and were subjected to pathway analysis. The intersection of all protein lists containing the respective higher reactive DIRAGs revealed 8 proteins (EEF1A1, HBP1, TXNDC5, TPM3, c8orf33, ILF3, MGEA5, LTBP3, HLA-C and UBA1) higher reactive in seropositive RA, seronegative RA and PIA (Figure 3A, not corrected for duplicated proteins). Signal intensities of the top 10 proteins of each comparison are given as boxplots in Supplementary Materials Figure S2.

### 2.3. Reactome Pathway Analysis

At the time of analysis, Reactome version 66 (human), version 69 (rat) and version 77 (reference) were used. 206 significant DIRAGs derived from the human class comparison remained higher reactive in seropositive RA after gene symbol cleansing for unambiguous IDs, 101 of them were found in Reactome (560 pathways were hit by them).

Reactome analysis elucidated 25 pathways with an FDR ≤ 0.1—these pathways are listed in Supplementary Materials Table S1. All of the top 25 pathways showed high significance for their respective entities *p*-value (0.004–10^−10^), the top 16 pathways preserved false discovery rates (FDRs) < 0.05 after Benjamini-Hochberg (BH) correction. Comparison of the pathway’s respective rank within the reference analysis indicates the pathways Endosomal/Vacuolar pathway and Antigen Presentation: Folding, assembly and peptide loading of class I MHC as overrepresented (entities *p*-values 7.16 × 10^−11^ and 0.004, FDR: 0.981). HLA-C was the associated gene for these two pathways which was also involved in eight additional enriched pathways. The synopsis of the genes/proteins with a bearing role within the pathway analysis is given in Table 1.

For seronegative RA, 147 higher reactive DIRAGs versus seropositive RA out of the 221 cleaned IDs were found in Reactome which hit 812 pathways. Fourteen (14) of them showed high significance from 10^−4^–10^−16^ (overall range 0.014–1.11 × 10^−16^) and preserved significance (*p* < 0.05) after BH-correction. Without respect to the overrepresentation analysis, 10 identical pathways were elucidated in the top 25 for both disease serotypes (Supplementary Materials Tables S1 and S2).

For the PIA model 673 out of 988 cleansed DIRAGs with unambigious genesymbols were found in Reactome with 1397 hit pathways, the top 25 are given in Supplementary Materials Table S3. All of them showed high significance levels after multiple testing correction (2.08 × 10^−12^–0.002). The intersection of the top 25 pathways for seropositive RA, seronegative RA and PIA pathway analysis revealed 8 common pathways: the endosomal/vacuolar pathway, the antigen presentation: folding, assembly and peptide loading of class I MHC, interferon alpha/beta signaling, the ER-Phagosome pathway, the interferon pathway, antigen processing–cross presentation, interferon gamma signaling and cytokine signaling in the immune system with preserved significance after BH-correction (Supplementary Materials Tables S1–S3). This suggests similarities of the auto-antibody signatures of RA and PIA and hence the disease reflection in this animal model which shows many clinical features of RA.

**Figure 4:**
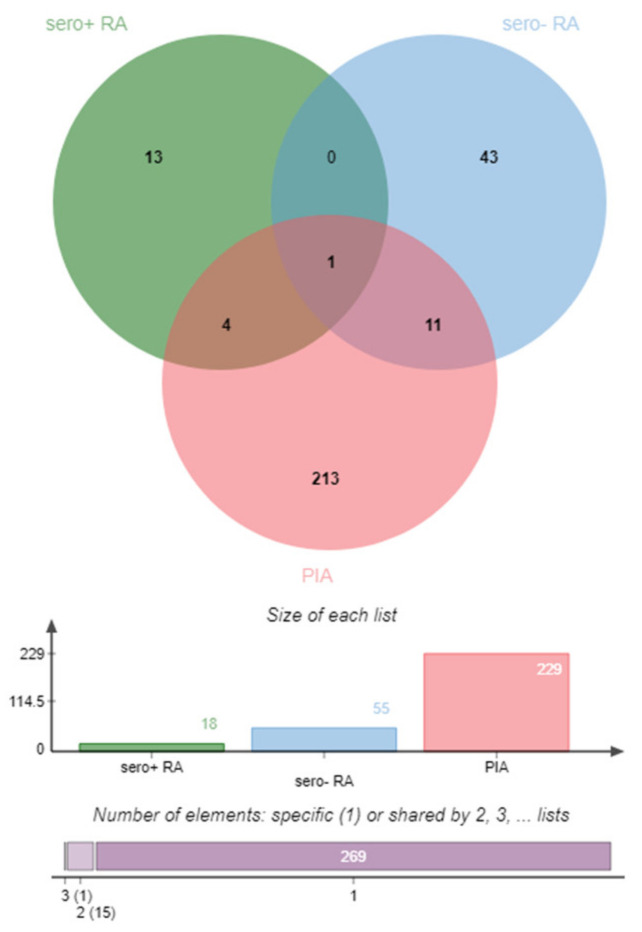
Graphic representation (Venn diagram) of the involved genes of the top 25 pathways for the comparisons: seropositive RA vs. seronegative RA (seropos, red), seronegative RA vs. seropositive RA (blue) and 7- vs. 24-day PIA corrected for controls (PIA, green). Venn diagram created with JVenn [9].

Found gene symbols of the top 25 pathways for each comparison were intersected for overlapping features (Figure 4). This intersection of the gene lists showed HLA-C as single common higher reactive DIRAG for the seropositive, seronegative RA and the PIA model. The candidate role of HLA-C as important player in rheumatic diseases was recently reviewed by Siegel et al. Besides HLA-C, GBP6, EIF4G2 and HNRPDL were identified between seropositive RA and PIA. Between seronegative RA and PIA, 11 overlaps were identified: HLA-A, FLNA, CCND1, FN1, APEH, VCL, NUP62, LCP1, PSMC4, DDOST and EEF1A1. Previously described disease involvement of genes is given in Table 1.

Based on the comparison of elucidated DIRAGs with “biological relevance” (involved in pathway analysis), CCND1 and PSMC4 arose as potential novel autoantibodies/autoantigens since they are herein described for the first time. Other DIRAGs such as MSN and NUP62 were extensively described as autoantigens in systemic auto-immune diseases.

All of the overlapping pathways between human RA and PIA are linked to intra-cellular protein degrading and signaling processes, and hence, antigen processing and presentation processes. Seropositive versus seronegative RA showed significant pathways for the intra-cellular skeleton and transport system (seropositive RA). In seronegative RA, pathways for the adaptive immune response and transcriptional regulation were found significant. Besides antigen processing and presentation pathways, the PIA model showed significant pathways for transcription, translation, RNA metabolism and processing.

### 2.4. WebGestalt Pathway Analysis

**Figure 5:**
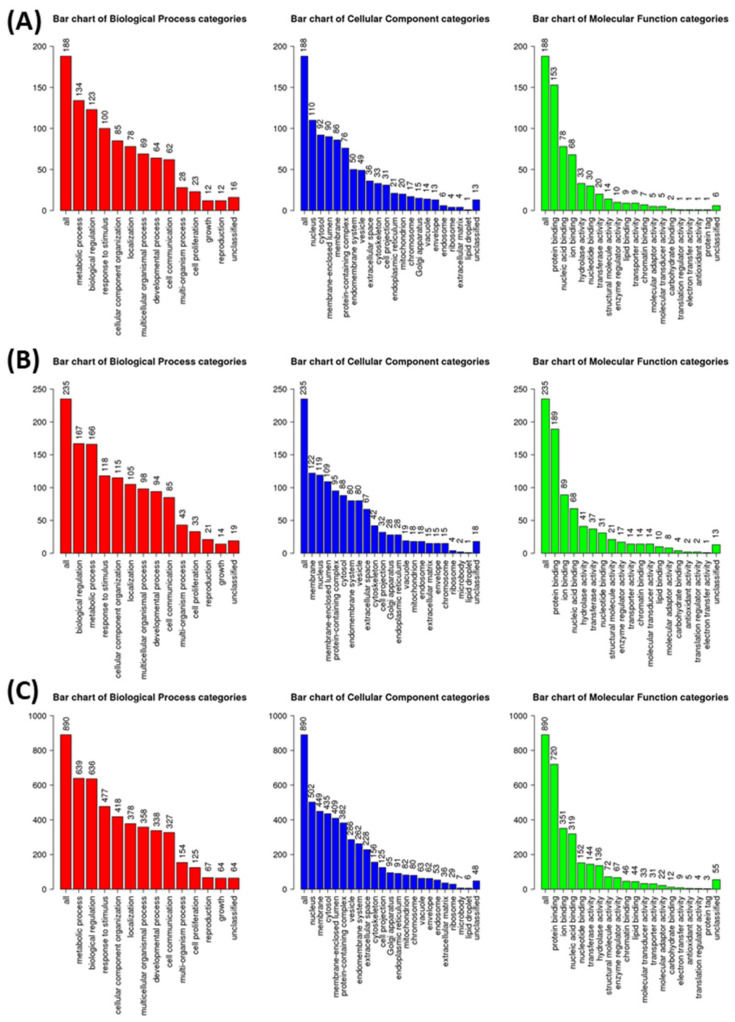
GOslim summaries for DIRAGs identified as higher reactive in (**A**) seropositive RA versus seronegative RA, (**B**) seronegative RA vs. seropositive RA and (**C**) PIA animals 7 and 24 days after disease induction corrected for signatures of control animals.

WebGestalt analysis was appended for GeneOntology (GO) annotation of elucidated DIRAGs based on their respective gene symbols. For seropositive RA from *n* = 206 higher reactive DIRAGs with cleansed gene symbols, 130 could be annotated for functional categories. For seronegative RA from *n* = 261, higher reactive DIRAGs with cleansed gene symbols, 156 were mapped to functional categories. From the PIA list, *n* = 605 could be annotated for functional categories. From the 16k annotations (reference list), *n* = 3246 IDs were mapped to functional categories. The GOSlim summaries for the biological processes, cellular components and molecular functions categories are given in Figure 5. Results for the top 10 pathways with the respective identified gene symbols are compiled in Table 2, Table 3 and Table 4. GeneSymbol lists containing the gene symbols of suspected biological relevance (included in the top 10 gene sets) were intersected (Supplementary Materials), whereby EEF1A1 arose as single hit between all subjected gene sets. The GOSlim summaries of the annotated gene symbols show similar rankings for the associated categories, with a slightly different order for seronegative RA (biological regulation followed by metabolic processes, vice versa in seropositive RA).

### 2.5. Correlation with Clinical Disease Activity Index (CDAI)

**Figure 6:**
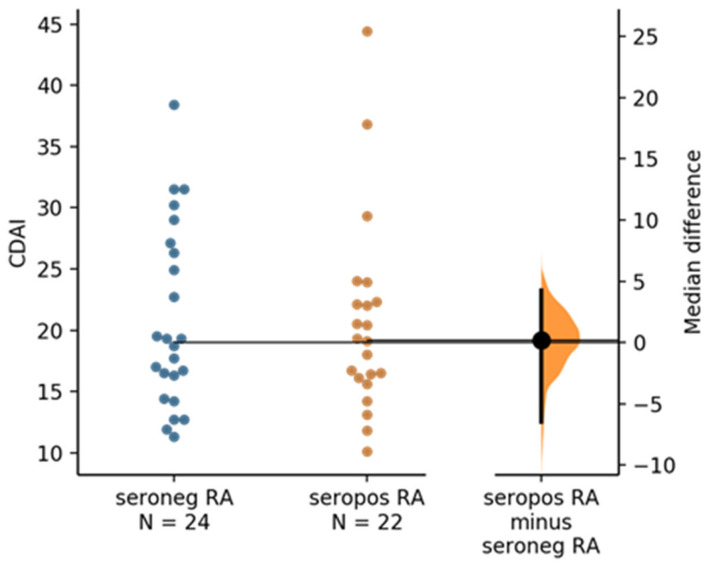
The median difference between seronegative RA and seropositive RA is shown in the above Gardner–Altman estimation plot. Both groups are plotted on the left axes; the mean difference is plotted on a floating axis on the right as a bootstrap sampling distribution. The mean difference is depicted as a dot; the 95% confidence interval is indicated by the ends of the vertical error bar. The unpaired median difference between seronegative RA and seropositive RA is 0.2 [95.0%CI −6.45, 4.25]. The *p* value of the two-sided permutation *t*-test is 0.9 (calculation and plot generated by https://www.estimationstats.com (accessed on 30 December 2021) according [41].

Clinical disease activity scores (CDAI) were available for 46 of the total 48 RA samples (*n* = 22 seropositive and *n* = 24 seronegative RA samples). Estimation statistics did not show any significant difference in CDAI when comparing seropositive versus seronegative RA (Figure 6).

To investigate if the antibody reactivities of the 46 human RA samples (for which CDAI was available) are correlated with the clinical disease activity, a quantitative trait analysis of the human RA sample data was conducted with the clinical disease activity index (CDAI) as quantitative trait (BRB ArrayTools). In total, 429 different antigenic proteins showed a significant correlation of *r* = ±0.29–±0.49 (*p* < 0,05; Spearman’s rank correlation) with disease activity. The *n* = 153 positively correlated antigens (*r* = 0.29–0.46) as well as 276 negatively correlated antigens (*r* = −0.29–−0.49) where then subjected to the Reactome pathway browser.

Pathways elucidated for the proteins positively correlated with CDAI showed RNA Polymerase I Transcription Initiation, RUNX1 regulates expression of components of tight junctions and Metabolism of RNA as the top three hits (*p* < 0,05; FDR 3.37 × 10^−1^, the latter not shown; details giving in Supplementary Materials Table S4A). Exemplarily, the involvement of the DIRAGs within the Metabolism of RNA (super-)pathway is given in Supplementary Materials Figure S3A.

Reactome analysis of the negatively correlated antigens displays a completely divergent panel of pathways compared to the those of positively correlated antigens. The top five of these pathways are: Antigen Presentation: Folding, assembly and peptide loading of class I MHC, Endosomal/Vacuolar pathway, Class I MHC mediated antigen processing & presentation, ER-Phagosome pathway and Interferon Signaling (*p* = 1.11 × 10^−16^; FDR = 1.57 × 10^−14^; Supplementary Materials Table S4B and Figure S3B exemplifying the Class I MHC mediated antigen processing & presentation-pathway as extracted from Reactome pathway browser). However, these pathways of the positively correlated antigens resemble with those found enriched for the DIRAGS from the other contrasts e.g., “seropositive versus seronegative RA”, “higher reactive in seronegative RA” and “higher reactive in established PIA” (Supplementary Materials Tables S1–S3). As an example, the involvement of the DIRAGs within the Antigen Presentation (super-)pathway is given in Supplementary Materials Figure S4B.

**Table 2:** Top 10 identified gene sets in the WebGestalt analysis for DIRAGs higher reactive in seropositive (A) and seronegative RA (B) and PIA animals (C) during the disease onset period (24 days after pristane induction). The Reactome GeneSet and link to the Pathway browser, gene set description, the respective *p*-value and the GeneSymbols of significantly higher reactive DIRAGs are given.

(A) DIRAGs Higher Reactive in Seropositive RA (Seropositive vs. Seronegative RA)			
GeneSet (Reactome)	Description	*p*-Value	Gene Symbol
R-HSA-936440	Negative regulators of DDX58/IFIH1 signaling	0.0039	UBA7, CYLD, ISG15, PCBP2
R-HSA-202403	TCR signaling	0.0039	VASP, LAT, PTPRC, PSME4, NFKB1, ITK, PSMD13, PSMB10
R-HSA-6790901	rRNA modification in the nucleus and cytosol	0.0070	NOP2, TBL3, UTP14A, RRP9, IMP4
R-HSA-202433	Generation of second messenger molecules	0.0106	VASP, LAT, ITK
R-HSA-8953854	Metabolism of RNA	0.0114	NOP2, PHAX, EIF4A3, EIF4G1, TBL3, SF1, RPL4, THOC3, UTP14A, EXOSC10, TSEN54, PPP2R1A, DDX42, DCP1A, PSME4, SF3B5, RRP9, PUS3, PSMD13, SF3A1, PSMB10, IMP4, PCBP2
R-HSA-1660662	Glycosphingolipid metabolism	0.0134	ESYT1, ESYT2, SUMF2
R-HSA-168249	Innate Immune System	0.0143	EEF1A1, TXNDC5, SDCBP, PRKCSH, LAT, STAT6, UBA7, CYLD, PTPRC, PPP2R1A, IQGAP1, PSME4, CYB5R3, NFKB1, ITK, CYFIP2, HLA-C, DPP7, PSMD13, VAV2, ELMO2, PSMB10, PDAP1, ISG15, PCBP2
R-HSA-352230	Amino acid transport across the plasma membrane	0.0147	SLC7A5, SLC3A2
R-HSA-168928	DDX58/IFIH1-mediated induction of interferon-alpha/beta	0.0148	UBA7, CYLD, NFKB1, ISG15, PCBP2
R-HSA-381183	ATF6 (ATF6-alpha) activates chaperone genes	0.0215	ATF4, NFYA

**Table 3:** Top 10 identified gene sets in the WebGestalt analysis for DIRAGs higher reactive in seropositive (A) and seronegative RA (B) and PIA animals (C) during the disease onset period (24 days after pristane induction). Reactome GeneSet and link to the Reactome pathway browser, gene set description, the respective *p*-value and GeneSymbols of significantly higher reactive DIRAGs are given.

(B) DIRAGs Higher Reactive in Seronegative RA (Seropositive vs. Seronegative RA)			
GeneSet (Reactome)	Description	*p*-Value	Gene Symbol
R-HSA-74217	Purine salvage	0.0010	AMPD2, APRT, HPRT1
R-HSA-8956321	Nucleotide salvage	0.0051	AMPD2, APRT, HPRT1
R-HSA-6798695	Neutrophil degranulation	0.0058	APEH, IMPDH2, APRT, STK10, TXNDC5, DDOST, HLA-C, CTSD, SPTAN1, C3, EEF1A1, TCIRG1, VCL, DYNC1H1, PSMC3, DSP, GUSB, CCT8
R-HSA-1474244	Extracellular matrix organization	0.0072	LTBP3, TGFB1, LAMC1, COL1A2, HSPG2, CTSD, SERPINH1, ADAMTS4, ADAM19, PLOD1, ITGA3, COMP
R-HSA-8941856	RUNX3 regulates NOTCH signaling	0.0074	JAG1, NOTCH1, KAT2A
R-HSA-8878159	Transcriptional regulation by RUNX3	0.0150	JAG1, PSMC5, TGFB1, NOTCH1, CCND1, KAT2A, PSMC3
R-HSA-5688426	Deubiquitination	0.0186	OTUB1, USP30, PSMC5, TADA2B, TGFB1, ACTB, KAT2A, UIMC1, MBD6, PSMC3, AXIN1, RAD23A
R-HSA-425393	Transport of inorganic cations/anions and amino acids/oligopeptides	0.0218	SLC4A2, SLC1A5, SLC20A2
R-HSA-3000178	ECM proteoglycans	0.0243	TGFB1, LAMC1, COL1A2, HSPG2, COMP
R-HSA-5663202	Diseases of signal transduction	0.0253	JAG1, PSMC5, CUX1, TGFB1, NOTCH1, ACTB, POLR2G, KAT2A, MTOR, HDAC6, VCL, LCK, PSMC3, AXIN1

**Table 4:** Top 10 identified gene sets in the WebGestalt analysis for DIRAGs higher reactive in seropositive (A) and seronegative RA (B) and PIA animals (C) during the disease onset period (24 days after pristane induction). Reactome GeneSet and link to Reactome pathway browser, gene set description, the respective *p*-value and GeneSymbols of significantly higher reactive DIRAGs are given.

(C) DIRAGs Higher Reactive in PIA Animals (Corrected for DIRAGs Higher Reactive in PBS Animals, PIA vs. Control Animals)			
GeneSet (Reactome)	Description	*p*-Value	Gene Symbol
R-HSA-156827	L13a-mediated translational silencing of Ceruloplasmin expression	1.31 × 10^−5^	RPL7, RPL17, RPL27A, EIF4B, EIF4H, EIF4G1, EIF3A, RPS10, RPL10A, RPL26, RPS25, RPL41, RPL4, RPL24, RPS19, EIF4E, RPS18, EIF3H, RPL12, RPS4Y2, RPL22, RPL15, RPS5, RPL27, EIF3M, EIF3G, EIF3B
R-HSA-72706	GTP hydrolysis and joining of the 60S ribosomal subunit	1.31 × 10^−5^	RPL7, RPL17, RPL27A, EIF4B, EIF4H, EIF4G1, EIF3A, RPS10, RPL10A, RPL26, RPS25, RPL41, RPL4, RPL24, RPS19, EIF4E, RPS18, EIF3H, RPL12, RPS4Y2, RPL22, RPL15, RPS5, RPL27, EIF3M, EIF3G, EIF3B
R-HSA-72613	Eukaryotic Translation Initiation	1.54 × 10^−5^	RPL7, RPL17, RPL27A, EIF4B, EIF2B4, EIF4H, EIF4G1, EIF3A, RPS10, RPL10A, RPL26, RPS25, RPL41, RPL4, RPL24, RPS19, EIF4E, RPS18, EIF3H, RPL12, RPS4Y2, RPL22, RPL15, RPS5, RPL27, EIF3M, EIF3G, EIF3B
R-HSA-72737	Cap-dependent Translation Initiation	1.54 × 10^−5^	RPL7, RPL17, RPL27A, EIF4B, EIF2B4, EIF4H, EIF4G1, EIF3A, RPS10, RPL10A, RPL26, RPS25, RPL41, RPL4, RPL24, RPS19, EIF4E, RPS18, EIF3H, RPL12, RPS4Y2, RPL22, RPL15, RPS5, RPL27, EIF3M, EIF3G, EIF3B
R-HSA-72689	Formation of a pool of free 40S subunits	7.14 × 10^−5^	RPL7, RPL17, RPL27A, EIF3A, RPS10, RPL10A, RPL26, RPS25, RPL41, RPL4, RPL24, RPS19, RPS18, EIF3H, RPL12, RPS4Y2, RPL22, RPL15, RPS5, RPL27, EIF3M, EIF3G, EIF3B
R-HSA-156842	Eukaryotic Translation Elongation	1.00 × 10^−4^	RPL7, RPL17, RPL27A, RPS10, EEF1D, RPL10A, RPL26, RPS25, RPL41, RPL4, RPL24, RPS19, RPS18, RPL12, EEF1G, EEF1A1, RPS4Y2, RPL22, RPL15, RPS5, RPL27
R-HSA-72766	Translation	2.21 × 10^−4^	PPA1, VARS, RPL7, MRPL54, RPL17, RPL27A, SARS, EIF4B, EIF2B4, LARS, EIF4H, EIF4G1, AURKAIP1, YARS, EIF3A, DDOST, APEH, RPS10, EEF1D, RPL10A, RPL26, FARSA, HARS, RPS25, RPL41, RPL4, RPL24, PARS2, RPS19, EIF4E, AARS2, RPS18, EIF3H, RPL12, MRPS6, EEF1G, OXA1L, EEF1A1, RPS4Y2, RPL22, RPL15, RPS5, RPL27, EIF3M, EIF3G, EIF3B
R-HSA-72702	Ribosomal scanning and start codon recognition	2.33 × 10^−4^	EIF4B, EIF4H, EIF4G1, EIF3A, RPS10, RPS25, RPS19, EIF4E, RPS18, EIF3H, RPS4Y2, RPS5, EIF3M, EIF3G, EIF3B
R-HSA-927802	Nonsense-Mediated Decay (NMD)	2.48 × 10^−4^	SMG5, RPL7, RPL17, RPL27A, EIF4G1, RPS10, RPL10A, RPL26, RPS25, RPL41, RPL4, RPL24, RPS19, SMG8, RPS18, RPL12, SMG7, UPF1, RPS4Y2, RPL22, RPL15, RPS5, RPL27
R-HSA-975957	Nonsense Mediated Decay (NMD) enhanced by the Exon Junction Complex (EJC)	2.48 × 10^−4^	SMG5, RPL7, RPL17, RPL27A, EIF4G1, RPS10, RPL10A, RPL26, RPS25, RPL41, RPL4, RPL24, RPS19, SMG8, RPS18, RPL12, SMG7, UPF1, RPS4Y2, RPL22, RPL15, RPS5, RPL27

## 3. Discussion and Conclusions

The presence of auto-antibodies serves as one of the European League Against Rheumatism (EULAR) classification criteria for rheumatoid arthritis [42]. The serological status for rheumatoid factor (RF) and anti-citrullinated protein antibodies (ACPAs) allows stratification into subgroups of seropositive and seronegative RA. It is widely accepted and discussed that autoantibodies are of predictive value for the development of RA and the severeness of the disease outcome. However, the role of auto-antibodies within the disease pathogenesis is not completely understood. The feasibility of autoantibody based pathway analysis was reported in previous work [43,44,45]. 

Within the presented analysis we conducted IgG profiling of treatment-naive plasma samples from RA patients and PIA animals on high density protein microarrays. Isolated IgG from 96 human plasma samples and 8 animals was probed on AIT’s 16k protein microarray and identified DIRAGs subjected to pathway analysis using Reactome pathway browser and the WebGestalt toolkit to elucidate involved pathways. The identification of autoantibody signatures could not only aid in improving the early diagnosis of systemic autoimmune diseases, but also provide insights into the role of autoantibodies in the disease pathogenesis. Therefore, pathway analysis of DIRAGs was conducted in RA and compared to an established rodent model of RA to investigate the similarity of obtained immune signatures. Differential reactivity analysis of IgG from seropositive versus seronegative RA samples resulted in 206 significantly higher reactive DIRAGs with unambiguous gene symbols in seropositive RA and 221 DIRAGs in seronegative RA. In PIA, 988 significant DIRAGs (*p* < 0.05) upon correction for control animals were revealed. EEF1A1, HBP1, TXNDC5, TPM3, c8orf33, ILF3, MGEA5, LTBP3, HLA-C and UBA1 were significantly (*p* < 0.05) higher reactive (FC > 1.35) in seropositive, seronegative RA and PIA.

Gene sets were separately subjected to pathway analysis using the Reactome pathway browser, the complete 16k gene list was used as reference. The top 25 significant pathways showed 10 overlapping pathways between seropositive and seronegative RA with high significance (*p* < 0.003), and 7 retained high significance after multiple testing correction (Benjamini-Hochberg (BH) procedure). The remaining 3 pathways preserved high significance in seronegative RA after correction for multiple testing (BH procedure) (Cytokine signaling in Immune System, Immunoregulatory interactions between lymphoid and a non-lymphoid cell and Class I MHC-mediated antigens processing and presentation). For PIA, the 7 highly significant pathways overlapping between seropositive and seronegative RA were found within the top 10 pathways with high significance after BH correction. Cytokine signaling in the immune system retained high significance after BH correction for the PIA analysis. The intersection of the gene lists of the found genes in the top 25 pathways revealed 16 genes of suspected biological relevance (HLA-C, GBP6, EIF4G2, MSN, HNRPDL, HLA-A, FLNA, CCND1, FN1, APEH, VCL, NUP62, LCP1, PSMC4, DDOST and EEF1A1). Two out of these, CCND1 and PSMC4, have previously not been described as (auto)antigens.

Aiming to counterbalance possible biases of the pathway analysis caused by redundant hits within the Reactome pathway browser analysis, WebGestalt analysis was appended. The major advantage of the WebGestalt tool within this study is the option of redundancy reduction which leads to identical results for the 16k microarray within the Reactome pathway browser (HLA was observed as the top hit within all pathway analyses, also for the reference design). Since seropositive and seronegative RA have distinct serological and radiological appearance and disease progression, different auto-antibody signatures for the serotypes could be plausible. This hypothesis, however, required the reduction of redundant database hits as the reference analysis showed the identical result. Within the WebGestalt analysis, no overlapping pathways for seropositive or seronegative RA and PIA were observed. EEF1A1 was observed as single hit between after intersection of the gene lists, which also arose within the Reactome pathway browser analysis. EEF1A1 was previously described as an autoantigen in type 1 diabetes by Koo et al. in 2014 [38] and is stably expressed in synovial fibroblast as shown by Schröder et al. in 2019 [39]. Furthermore, EEF1A1 is described as an autoantigen in Felty’s syndrome, which is a rare condition associated with RA encompassing splenomegaly and low neutrophil counts [40]. The clinical disease activity index (CDAI) of the investigated cohort comprised patients with moderate-to-severe disease activity status (CDAI mean 20.7 in seropositive RA and 21.9 in seronegative RA, compare Table 3). A correlation analysis with CDAI as quantitative trait showed *n* = 429 antigens with significant correlation between r = ±0,29–0,49 which in summary shows good correlation for CDAI and antibody reactivity. With regard to the treatment-naive status of considered patient samples, elucidated antigens and pathways should reflect disease characteristics free of effects of immune-modulatory treatments. Of interest, the pathways affected by our analysis of antigenic reactivities showing negative correlation are strongly associated with antigen presentation and immune regulation—these are statistically highly significant. This means an inverse relation and less autoantibody reactivity towards these self-antigens in mild or less-active disease with respect to the CDAI. On the contrary antigens functional in RNA-associated pathways (e.g., RNA Polymerase I Transcription Initiation, RUNX1 regulates the expression of components of tight junctions, Metabolism of RNA, etc.) showed positive correlation with disease activity– this means higher autoantibody reactivity towards self-antigens, were found in more severe or active disease.

Although these data need to be validated with an independent sample cohort, especially for those with positive correlation (when *p*-values were significant, but the false discovery rates are high, thus this interpretation has to be taken with caution), the negative correlation with disease activity goes in line with the same pathways relevant in antigen presentation and immune regulation, as found significant when comparing the seropositive vs. seronegative human RA and in the PIA rodent RA model the animals expressing RA disease vs. those before RA induction. 

To our knowledge, this is the first comprehensive comparative study of rodent and human autoantibody signatures in RA. Taken together, the pathways elucidated from autoantibody signatures underpin the previously described clinical similarities between RA and PIA, suggesting shared pathways in disease initiation and progression. Therefore we conclude that IgG profiling on high density protein microarrays offers (I) the possibility to reveal novel autoantigens for diagnostic or therapeutic applications and (II) gives insights into the role of auto-antibodies within the pathogenesis.

## 4. Materials and Methods

### 4.1. Samples

Plasma samples from 96 treatment naive human individuals were provided by the biobank of the Division of Rheumatology of the Medical University of Vienna. Determination of RF and ACPA status were determined as previously described [46] and RA patients stratified respectively to seropositive (RF and ACPA positive) and seronegative (RF and ACPA negative) RA. Samples were equally distributed (*n* = 24 per group) over the patient groups osteoarthritis, seropositive RA, seronegative RA and bone-erosive disease-free controls (healthy). The characteristics of the human cohort are given in Table 5.

Serum from 8 animals was provided by the Division of Rheumatology of the Medical University of Vienna. The animal cohort comprised serum from *n* = 3 control group rats and *n* = 5 immunized rats collected 5, 7 and 24 days after Pristane (2,6,10,14-Tetramethylpentadecan) or PBS treatment; this arthritis model (Pristane induced arthritis (PIA)) and its protocol was previously described elsewhere in detail [7].

### 4.2. Antibody Isolation

IgG was isolated with the Melon^TM^ Gel IgG Spin Purification Kit (Thermo Scientific^TM^ 45,206) from human and murine plasma by diluting 15 µL plasma with 95 µL of purification buffer and isolation according manufacturer’s instructions. Antibody concentration was determined as the means of A280 duplicate measurements (Epoch Take3 system) and the integrity of antibodies was determined via gradient sodium dodecylsulfate (SDS) polyacrylamide gel electrophoresis (NuPAGE^TM^ 4–12% Bis-Tris (Invitrogen^TM^ NO0336) in 1X MOPS (Invitrogen^TM^ NP0050)) and subsequent Coomassie staining (Invitrogen^TM^ SimplyBlue^TM^ SafeStain). Two µg of eluate was mixed with 2.5 µL 4X LDS buffer (Pierce^TM^ 84788) and filled with buffer to 10 µL, denatured at 70 °C for 10 min and loaded to each lane and gel run at 180 V for 60 min. IgG was concentration adjusted to 0.3 mg/mL (human) and 0.2 mg/mL (murine) with the kit provided buffer and stored −20 °C until slide processing.

### 4.3. Protein Microarray Processing

AIT’s 16k protein microarray is an in-house printed, high density protein microarray derived from the UniPEx expression library. Production of recombinant proteins was previously described in detail elsewhere [44,47,48]. In brief, the array represents 5449 annotated human proteins in one or more *E. coli* cDNA clones (15,417 cDNA clones in total). Purified 6xHis-Tag proteins are spotted in duplicates onto SU8 epoxy coated glass slides with an Arrayjet Marathon Argus inkjet microarray instrument. Bovine serum albumin, human serum albumin, human IgG, crude *E. coli* lysate and elution buffer are spotted as controls. Each batch of printed slides is subjected to a qualification experiment as previously described by Coronell et al., and slides are vacuum sealed and stored at 4 °C until processing. Briefly, this qualification experiment comprises the reliability analysis of the platform to comprehend an individual’s antibody fingerprint by crosswise mixing of two samples and subsequent correlation analysis of the obtained antibody profiles when 97% of DIRAGs correlated (r = 0.5–1) with the mixing ratio [43].

16k protein microarray slides were equilibrated to room temperature, slides pretreated by incubation with 2% SDS at 70 °C for 10 min and blocked with DIG Easy Hyb^TM^ for 30 min at room temperature (RT) in one tank equipped with magnetic stirrers. Slides were washed three times in 1× PBS pH 7.4 0.1% Triton X-100 (PBST; Gibco^TM^ 70011044 and Merck X100) for 5 min each with stirring and rinsed with Milli-Q^®^ water. Blocked slides were spin dried at 900 rpm for 4 min and put in dust-free hybridization chambers (Agilent G253A). Thawn samples were diluted to a final concentration of 0.15 mg/mL with 2× PBST 6% skimmed milk powder (Maresi Fixmilch) and 400 µL of sample dilution applied to each gasket slide (Agilent G2534-60003), slides placed on top and chambers closed. Upon removal of air bubbles, chambers were placed into hybridization ovens and incubated for 4 h at RT with 12 rpm. After incubation chambers were opened, microarray slides arranged in glass carriers and washed three times in fresh PBST for 5 min each in glass tanks with stirring followed by a Milli-Q^®^ water rinse and spin drying by centrifugation at 900 rpm for min. Human IgG was detected with Alexa Fluor 647 goat anti-human IgG (Life technologies A21445) diluted 1:10,000 in PBST 3% milkpowder and incubation for 1 h in glass tanks with stirring at RT. Rat IgG was detected with Alexa Fluor 647 Goat anti-Rat IgG (H+L) (Thermo Scientific^TM^ A-21347) diluted 1:5000 in PBST 3% milk powder. Slides were finally washed three times with fresh PBST in glass tanks with stirring before a final Milli-Q^®^ water rinse and spin drying. Up to 72 slides were processed within 1 batch, therefore in total 3 batches were conducted.

### 4.4. Image Acquisition and Data Extraction

Processed and spin-dried slides were sorted in the slide insert and fluorescence images of arrays acquired by scanning at 220% PMT gain (human) and 200% PMT gain (rat) with a Tecan PowerScanner (excitation wavelength 635 nm, 10 µm resolution). Acquired TIFF images were loaded in GenePix Pro 7.0, the .gal file aligned and spots of low quality manually flagged. Fluorescence data was extracted as .gpr files. Visual inspection of raw intensities as boxplot revealed batch effects associated with experimental runs, hence ComBat normalization was applied for removal of these effects [49]. ComBat normalized data were subjected to median normalization and investigated via k-means clustering which assigned the samples to two clusters in accordance to the experimental batches. Therefore, the kMeans cluster was used as blocking variable for the class comparison analysis.

### 4.5. Preprocessing and Differential Reactivity Analysis

All preprocessing and data analysis steps were conducted with RStudio [50] and BRB ArrayTools [8]. Raw .gpr files were loaded in BRB ArrayTools. Median fluorescence values were corrected for local median background, flagged and low intensity (< 100 MFI) features removed, log2 transformed and normalized (human: ComBat and median with array 1 as reference [rat]). Differential reactivity analysis was conducted as class comparison analysis between the assigned case versus control classes with a nominal significance cutoff of *p* < 0.05. Murine samples were assigned to groups based on their sampling timepoint (5, 7 and 24 days after immunization). Tuncel et al. previously characterized the PIA model in depth [7], hence the interval between 7 and 24 days is defined as the disease onset period. Class comparison was conducted for these two timepoints for PIA and control samples, respectively. DIRAGs higher reactive in the PIA group were corrected for higher reactive DIRAGs in the control group before gene list cleaning and pathway analysis to adjust for phased natural fluctuations.

### 4.6. Reactome Pathway Analysis

The resulting protein lists were filtered for fold-change, and proteins showing higher reactivities in the *case* group were subjected to the Reactome pathway browser [51], actually their respective GeneSymbols. “Project to human” was selected in the Options column and analyzed. Results files were downloaded as .csv and .pdf reports saved for further analysis in Microsoft Excel. The complete protein list of the 16k microarray was subjected to Reactome as reference analysis. The respective rank of the observed pathway on the 16k array served as scale for overrepresentation (ORA) assessment. *p*-values of elucidated pathways were controlled via the Benjamini-Hochberg (BH) correction (false discovery rate [FDR]) [52].

### 4.7. WebGestalt Pathway Analysis

For GeneOntology analysis and refined ORA, protein lists containing the higher reactive DIRAGs in the respective *case* group were subjected to the WebGestalt toolkit (pathway and Reactome as functional database, minimum number of genes per category *n* = 5 and top 10 as respective significance level [53]). The complete list of gene symbols presented on the 16k array was used as a reference list. Report files were downloaded in the default format and further analysed in Microsoft Excel and the JVenn [9].

## Figures and Tables

**Table 1:** Relevance—with respect to the previously described involvements of genes or proteins, of higher reactive DIRAGs overlapping within the identified top 25 pathways of the class—comparisons: seropositive RA vs. seronegative RA (both directions, compare Supplementary Materials Tables S1–S3) and PIA vs. control animals.

GeneSymbol	SwissProt ID	Overlap	Described as…	Reference
HLA-C	P10321	seropos RA, seroneg RA, PIA	genetic involvement	Siegel 2019 [10]
			higher expressed in RA synovium	Xiao 2016 [11]
			auto-antibodies present (citrullinated)	Lo 2020 [12]
GBP6	Q6ZN66	seropos RA, PIA	higher expression in RA?	Roche mRNA patent
EIF4G2	P78344	seropos RA, PIA	involvement in OA (miRNA-197)	Gao 2020 [13]
			citrullinated antigen	Okazaki 2009 [14]
			auto antigen Sjörgens	Uchadi 2005 [15]
			higher expressed in RA synovium	Xiao 2016 [11]
MSN	P26038	seropos RA, PIA	potential RA autoantigen	Wagatsuma 1996 [16]
			potential psoriasis autoantigen	Maejima 2014 [17]
			autoantigen in Behcets	Hussain 2020 [18]
			autoantigen in acquired aplastic anemia	Takamatsu 2006 [19]
			autoantigen in MPO-ANCA associated vasculitis	Suzuki 2014 [20]
			autoantigen in Sjörgens	Zhang 2018 [21]
			autoantigen in anti-phospholipid syndrome	Lin 2015 [22]
HNRPDL	O14979	seropos RA, PIA	autoantigen in RA (citrullinated)	Marklein 2021 [23]
HLA-A	P04439	seroneg RA, PIA	genetic involvement	Raychaudhuri 2012 [24]
			auto-antibodies present (citrullinated)	Lo 2020 [12]
FLNA	P21333	seroneg RA, PIA	auto-antibodies present; involved in microbial immunity	Pianta 2017 [25]
			auto-antibodies present (citrullinated)	Lo 2020 [12]
			synovium	Biswas et al. 2013 [26]
CCND1	P24385	seroneg RA, PIA	n.a.	n.a.
FN1	P02751	seroneg RA, PIA	elevated levels in synovium	Scott 1981 [27]
			autoantigen in RA (citrullinated)	Beers 2012 [28]
APEH	P13798	seroneg RA, PIA	auto-antibodies present (citrullinated)	Lo 2020 [12]
VCL	P18206	seroneg RA, PIA	auto antigen in RA (citrullinated)	Heemst 2015 [29]
NUP62	P37198	seroneg RA, PIA	higher expressed in Psoriasis arthritis PBMCs	Batliwalla 2005 [30]
			autoantibodies in myositis	Senecal 2014 [31]
			autoantibodies in SLE	Meulen 2017 [32]
			autoantibodies in Vasculitis/Sjörgens combination (single case report)	Fuchs 2020 [33]
			autoantibodies in primary biliary cirrhosis (PBS)	Bogdanos 2011 [34]
			autoantibodies in Psoriasis Arthritis	Yuan 2019 [35]
LCP1	P13796	seroneg RA, PIA	mRNA classifier	Liu 2021 [36]
PSMC4	P43686	seroneg RA, PIA	n.a.	n.a.
DDOST	P39656	seroneg RA, PIA	higher expression in Type2 Diabetes	Gupta 2019 [37]
EEF1A1	P68104	seroneg RA, PIA	auto-antibodies present in Type1 Diabetes	Koo 2014 [38]
			used as reference gene for synovial fibroblasts	Schröder 2019 [39]
			Auto-antibodies present in Felty’s syndrome	Ditzel 2000 [40]

**Table 5:** Sample characteristics of the investigated human cohort: age, biological sex, rheumatoid factor (RF), ACPA (CCP+) status and disease activity as clinical disease activity status (CDAI) are given.

Characteristic		Seropositive RA	Seronegative RA	Healthy Controls	Osteoarthritis
age	range (years)	24.7–76.8	33.6–77.9	41–68	35–78
	mean (years)	54.3	58.9	52.5	60.9
sex	male (*n*)	9	7	8	5
	female (*n*)	15	17	16	19
RF+		*n* = 24	-	-	-
CCP+		*n* = 24	-	-	-
disease activity	range (CDAI)	10.1–44.4	11.9–38.4	-	-
	mean (CDAI)	20.7	21.9	-	-

## Data Availability

Protein array data are available on request.

## Supplementary Materials

The following supporting information can be downloaded online. Figure S1: SDS Page for plasma isolated IgG; Figure S2: Boxplots of top 10 DIRAGs in human RA and the PIA rat model; Figure S3: (A) Involvement of antigens with significant positive CDAI correlation in Metabolism of RNA pathway. (B) the top pathway of antigens with significant positive CDAI correlation: Class I MHC mediated antigen processing & presentation; Table S1: Top 25 enriched pathways identified for DIRAGs higher reactive in seropositive versus seronegative RA in human IgG profiling. Table S2: Top 25 pathways enriched pathways for DIRAGs higher reactive in seronegative RA. Table S3: Top 25 enriched pathways for DIRAGs higher reactive in established PIA. Table S4: The enriched pathways for antigens positively (A) and the top 20 negatively (B) correlated with CDAI.

## Sample Availability

Samples of the compounds are not available from the authors.
