# Supplementary material for: Functional Analysis of Autoantibody Signatures in Rheumatoid Arthritis
Source: Molecules. 2022 Feb 21;27(4):1452. doi: 10.3390/molecules27041452 (PMC8876797; doi:10.3390/molecules27041452)
Supplement: Supplementary file 1 [file molecules-27-01452-s001.zip › Supplementary Figure S1.pdf]

Supplementary Figure S1

Supplementary Figure S1: (A) SDS Page for plasma isolated IgG. 2 µg of sample were loaded to each lane of a precasted gel (NuPAGE^{TM} 4-12% Bis-Tris (Invitrogen^{TM} NO0336), run in 1X MOPS (Invitrogen^{TM} NP0050) for 1h at 180V for 60min and subsequently Coomassie stained (Invitrogen^{TM} SimplyBlue^{TM} SafeStain). (B) sample loading scheme, obtained IgG concentration after purification and respective group of the loaded sample are given.

(A)

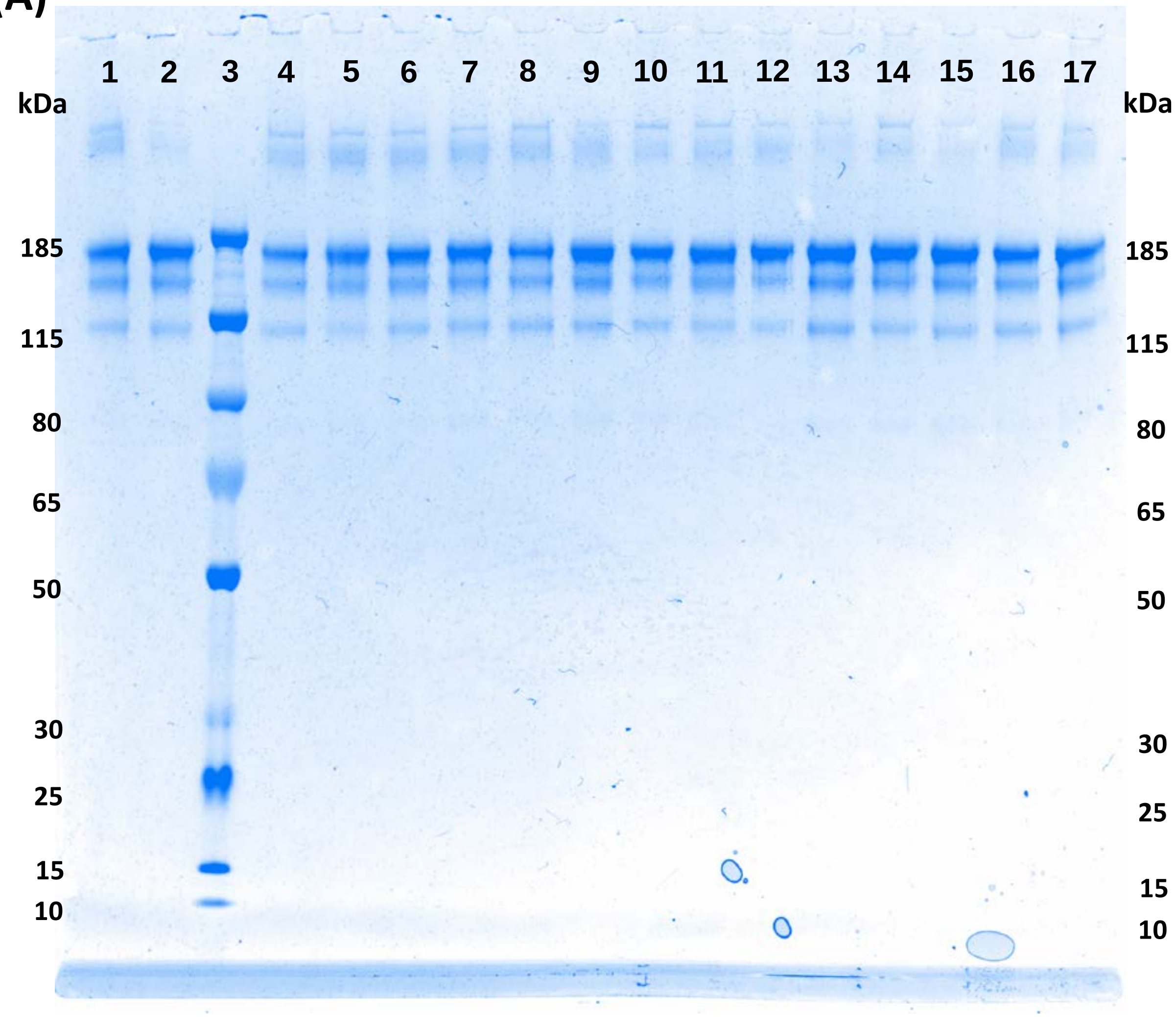

(B)

Lane	mg/mL	group
1	1.1	seronegative RA
2	1.1	healthy
3	ladder (NuPageRuler plus)	
4	1.03	osteoarthritis
5	1.02	healthy
6	2	healthy
7	1.03	seronegative RA
8	1.98	seropositive RA
9	1.02	seropositive RA
10	0.99	seronegative RA
11	0.99	healthy
12	1.07	seronegative RA
13	1.1	seropositive RA
14	1.01	osteoarthritis
15	1.09	healthy
16	1.02	seronegative RA
17	1.05	seropositive RA

2 µg sample loaded per lane
