# Supplementary material for: Functional Analysis of Autoantibody Signatures in Rheumatoid Arthritis
Source: Molecules. 2022 Feb 21;27(4):1452. doi: 10.3390/molecules27041452 (PMC8876797; doi:10.3390/molecules27041452)
Supplement: Supplementary file 1 [file molecules-27-01452-s001.zip › Supplementary Figure S2.pdf]

Supplementary Figure S2: Boxplots of top 10 DIRAGs in human RA and the PIA rat model

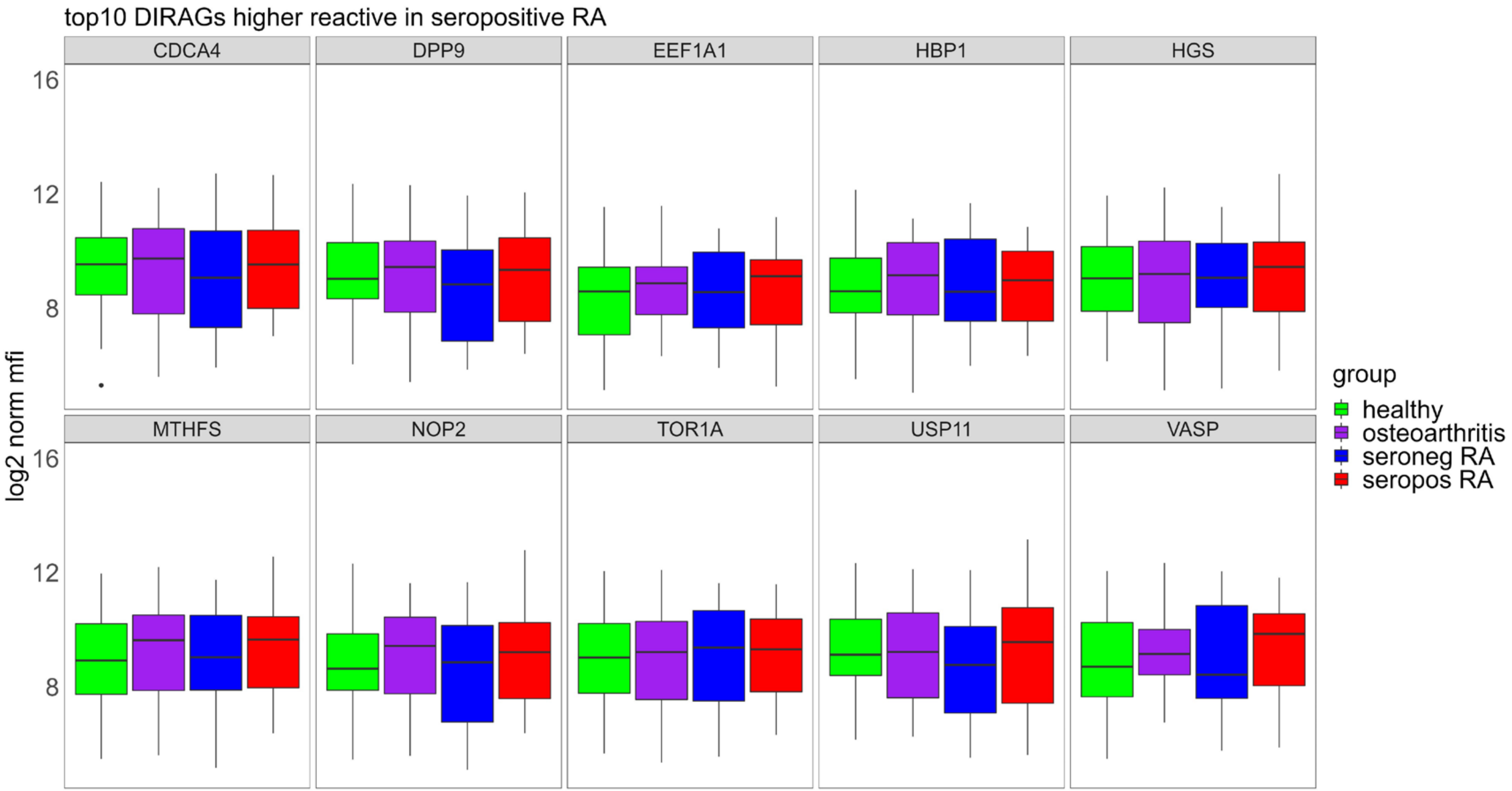

Supplementary Figure S2: signal intensities of the top10 DIRAGs significantly higher reactive in seropositive RA versus seronegative RA (sorted by p-value and fold-change). Each panel shows the groupwise intensities for healthy (green), osteoarthritis (purple), seronegative RA (blue) and seropositive RA (red).

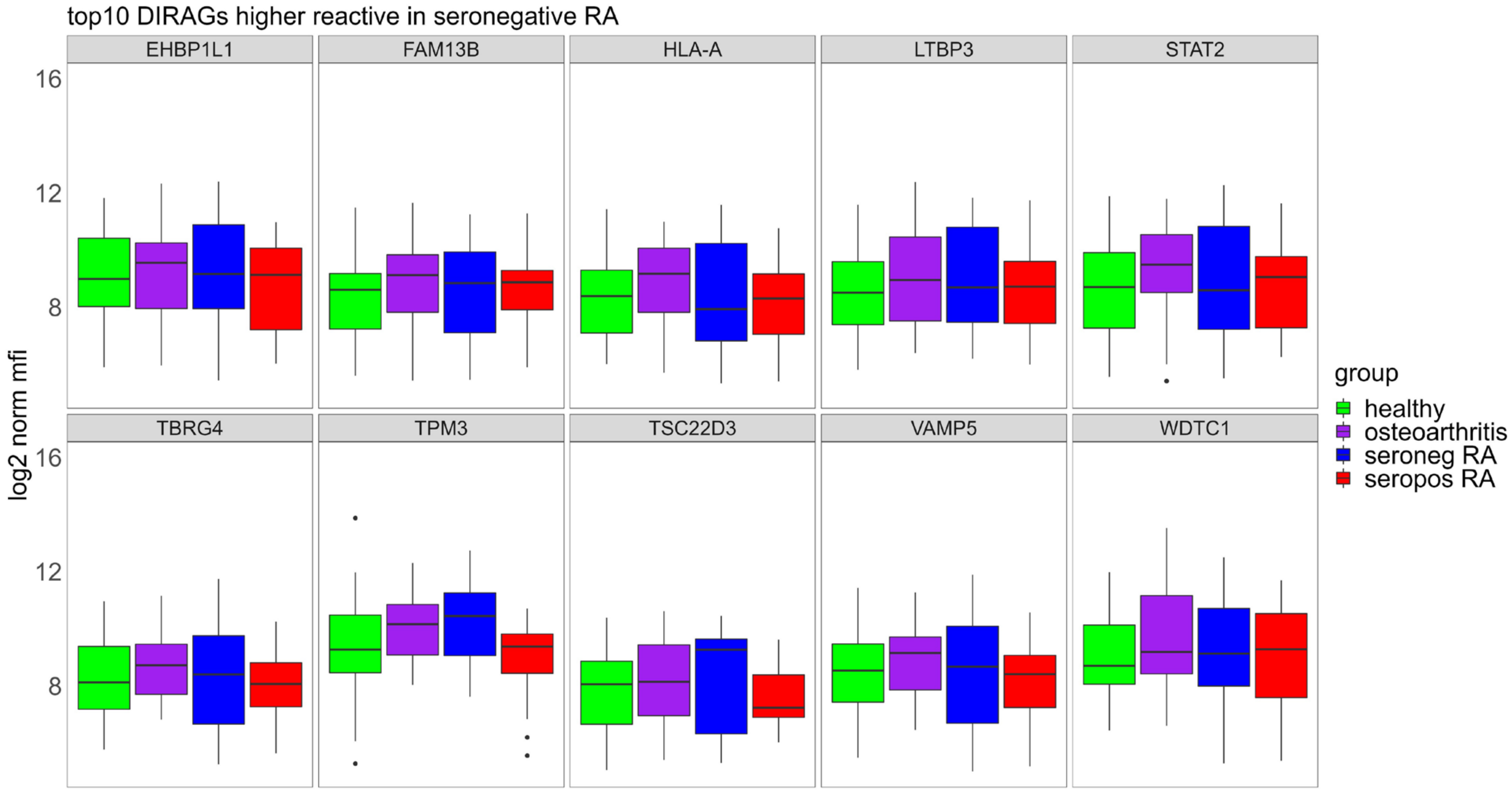

Supplementary figure 2: signal intensities of the top10 DIRAGs significantly higher reactive in seronegative RA versus seropositive RA (sorted by p-value and fold-change). Each panel shows the groupwise intensities for healthy (green), osteoarthritis (purple), seronegative RA (blue) and seropositive RA (red).

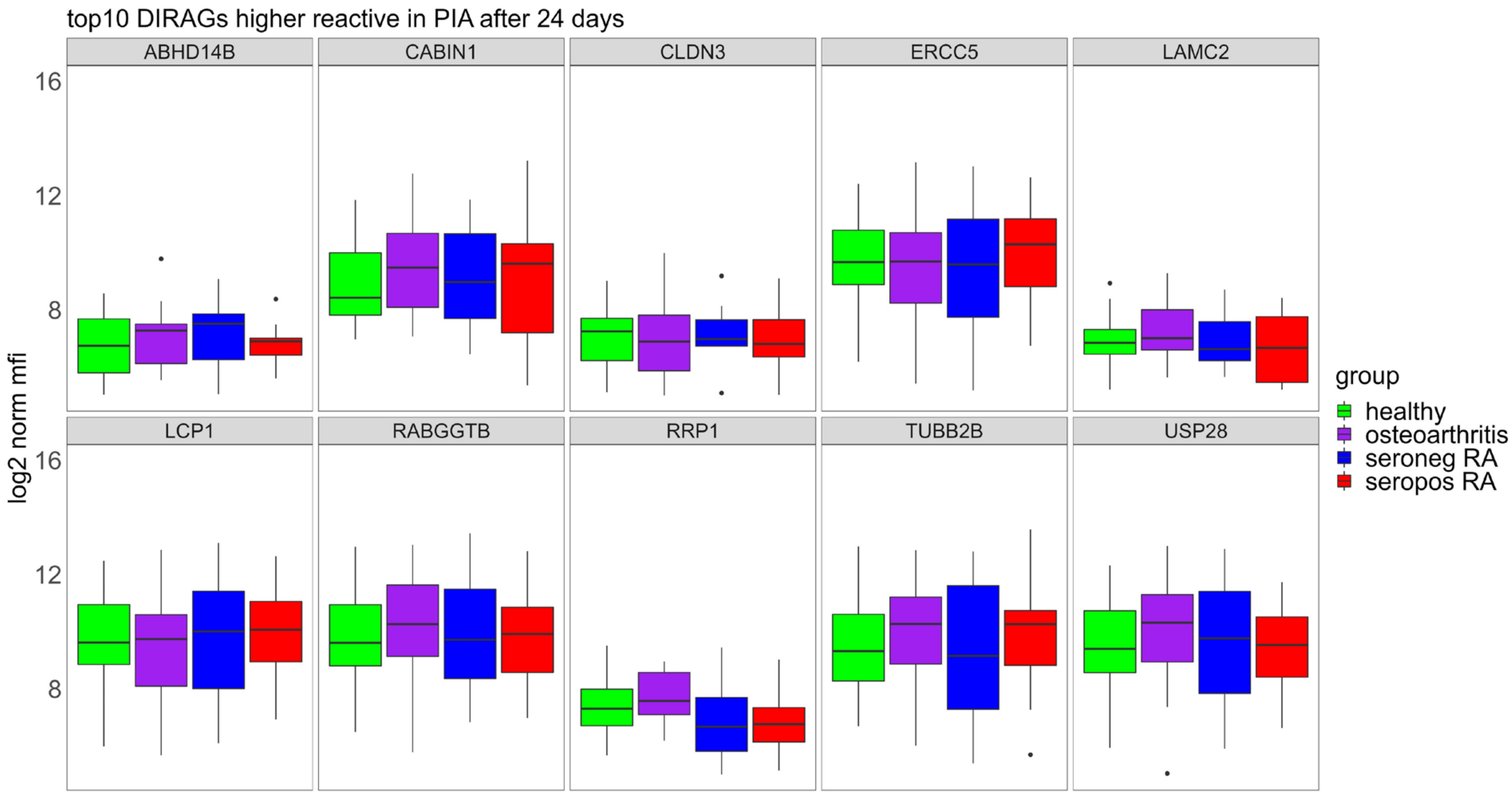

Supplementary figure 3: signal intensities of the top10 DIRAGs significantly higher reactive in PIA animals within the disease onset period (corrected for control animals, sorted by p-value and fold-change). Each panel shows the groupwise intensities for healthy (green), osteoarthritis (purple), seronegative RA (blue) and seropositive RA (red).
