# Supplementary material for: Functional Analysis of Autoantibody Signatures in Rheumatoid Arthritis
Source: Molecules. 2022 Feb 21;27(4):1452. doi: 10.3390/molecules27041452 (PMC8876797; doi:10.3390/molecules27041452)

Supplemental Figure S3: A) involvement of antigens with significant positive CDAI correlation in *Metabolism of RNA pathway* (for each of the sub-pathways, the number of hits and the respective FDR are given**)** as extracted from Reactome pathway browser [9].

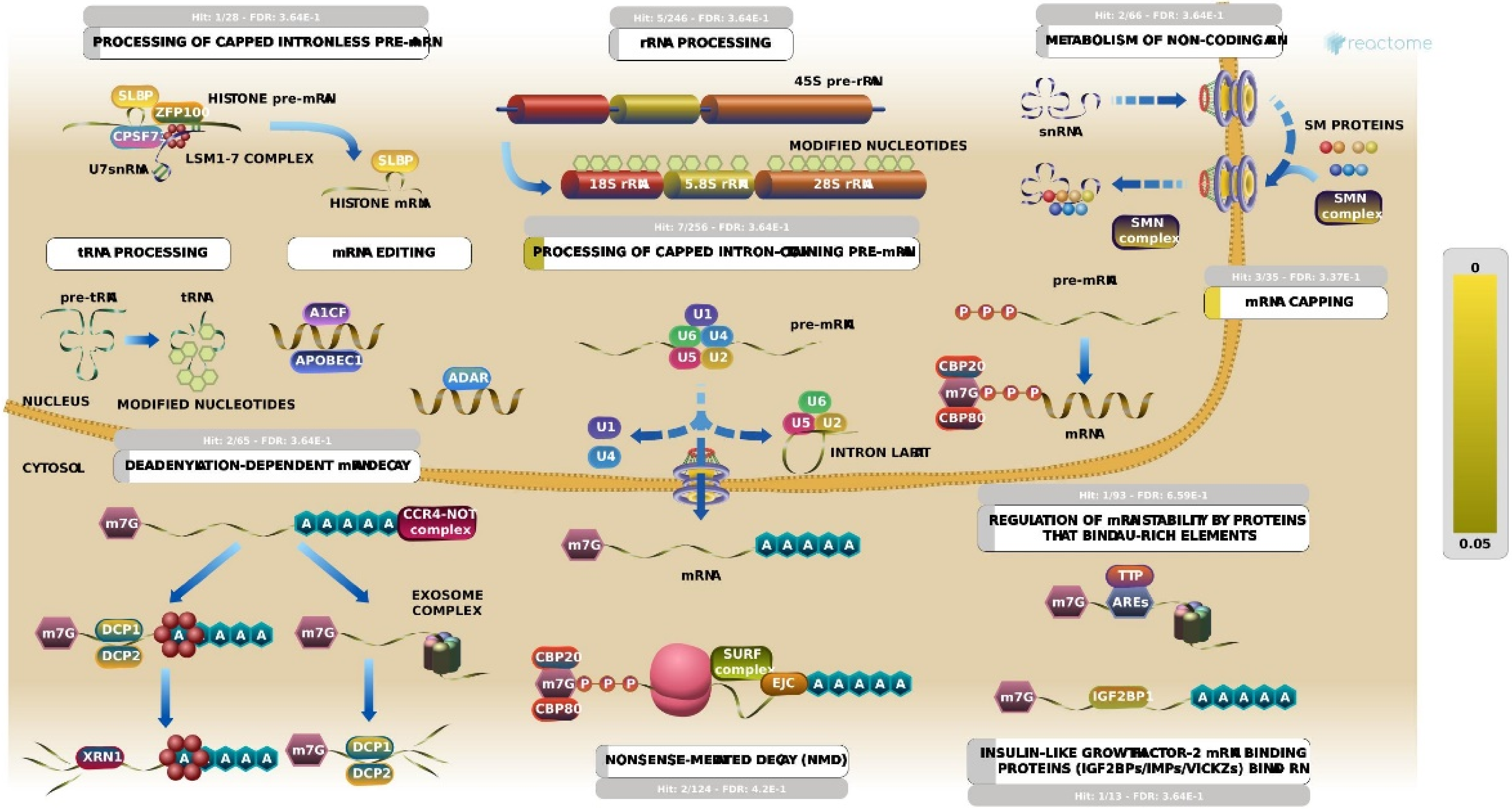

SUPPLEMENTAL Figure S3: B) the top pathway of antigens with significant positive CDAI correlation: *Class I MHC mediated antigen processing & presentation* as extracted from Reactome pathway browser [9].

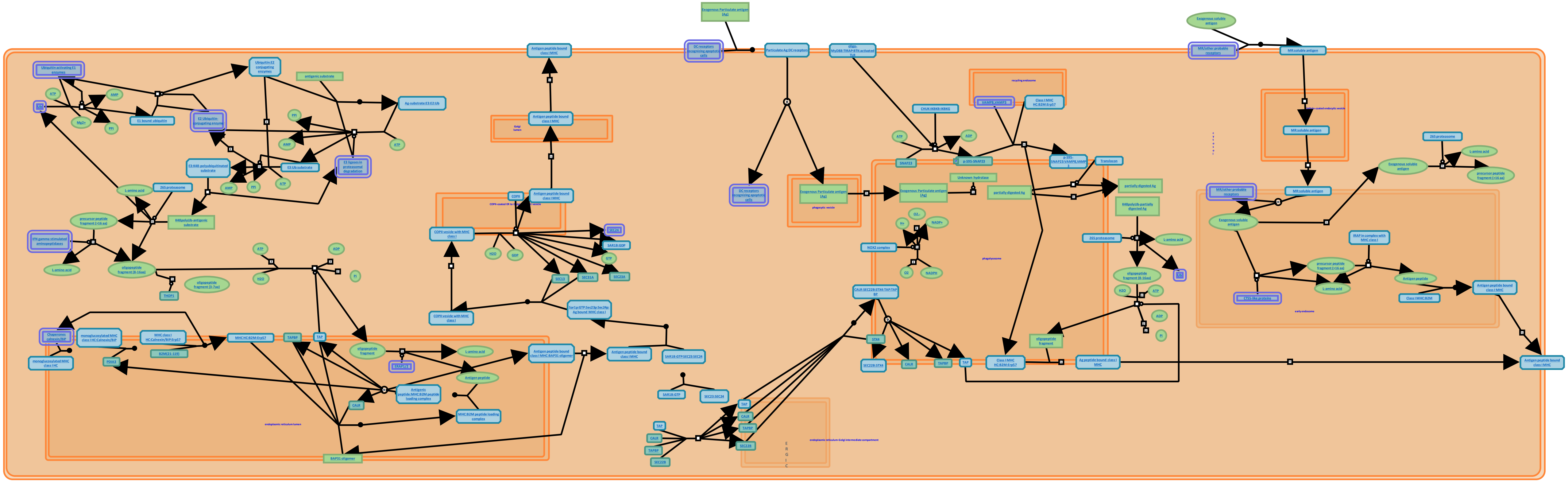

Supplement: Supplementary file 1 [file molecules-27-01452-s001.zip › Supplementary Figure S3.pdf]
