# Supplementary material for: Functional Analysis of Autoantibody Signatures in Rheumatoid Arthritis
Source: Molecules. 2022 Feb 21;27(4):1452. doi: 10.3390/molecules27041452 (PMC8876797; doi:10.3390/molecules27041452)
Supplement: Supplementary file 1 [file molecules-27-01452-s001.zip › Supplementary Table S1.pdf]

Supplementary Table S1

Supplementary Table S1: Top 25 enriched pathways identified for DIRAGs higher reactive in seropositive versus seronegative RA in human IgG profiling. Out of 206 clean genesymbols, 101 were found Reactome pathway browser. Name of the pathway (link to Reactome pathway browser), found and total entities, their respective p-value and FDR and found and total reactions are given. The genesymbol found in Reactome and the overrepresentation analysis of the complete 16k chip serving as reference for the respective pathway is given (continued on page 2).

Pathway name	found/total entities	entities p-value	entities FDR (BH)	found/total reactions	GeneSymbols found	found/total entities (16k)	rank (16k)
Endosomal/vacuolar pathway	14/82	$3.18 * 10^{-10}$	$1.99 * 10^{-7}$	3/4	HLA-C	74/82	1
Antigen Presentation: Folding, assembly and peptide loading of class I MHC	14/102	$4.98 * 10^{-9}$	$1.56 * 10^{-6}$	13/16	HLA-C	84/102	2
Interferon alpha/beta signaling	17/184	$3.64 * 10^{-8}$	$6.95 * 10^{-6}$	2/20	HLA-C, IRF5	120/186	41
ER-Phagosome pathway	16/164	$4.45 * 10^{-8}$	$6.95 * 10^{-6}$	4/10	HLA-C, PSMB11, PSMD14	122/173	3
Interferon gamma signaling	19/250	$1.16 * 10^{-7}$	$1.45 * 10^{-5}$	2/15	GBP6, HLA-C, IRF5	138/250	572
Antigen processing-Cross presentation	16/186	$2.38 * 10^{-7}$	$2.48 * 10^{-5}$	8/23	HLA-C, PSMB11, PSMD14	127/195	22
Interferon Signaling	21/392	$6.44 * 10^{-6}$	$5.74 * 10^{-4}$	6/66	EIF4G2, GBP6, HLA-C, IRF5, KPNA3	205/394	1102
Carboxyterminal post-translational modifications of tubulin	7/52	$4.17 * 10^{-5}$	0.003	6/6	TUBA1B, TUBB2B	15/55	1589
RHO GTPases activate IQGAPs	6/36	$4.65 * 10^{-5}$	0.003	5/5	IQGAP2, TUBA1B, TUBB2B	17/36	577
Microtubule-dependent trafficking of connexons from Golgi to the plasma membrane	5/22	$4.79 * 10^{-5}$	0.003	1/2	TUBA1B, TUBB2B	9/22	693
Transport of connexons to the plasma membrane	5/23	$5.89 * 10^{-5}$	0.003	1/3	TUBA1B, TUBB2B	9/23	761
Recycling pathway of L1	6/55	$4.50 * 10^{-4}$	0.023	6/14	MSN, TUBA1B, TUBB2B	30/55	368

The role GTSE1 in G2/M progression after G2 checkpoint	7/83	$7.00 * 10^{-4}$	0.034	4/10	PSMB11, PSMD14, TUBA1B, TUBB2B	53/83	87
Gap junction assembly	5/41	$8.24 * 10^{-4}$	0.036	3/16	TUBA1B, TUBB2B	11/41	1485
Post-chaperonin tubulin folding pathway	4/25	0.001	0.042	9/9	TUBA1B, TUBB2B	11/25	610
Immunoregulatory interactions between a lymphoid and a non-lymphoid cell	14/316	0.001	0.056	7/43	HLA-C	95/316	2008
HSP90 chaperone cycle for seroid hormone receptors (SHR)	6/70	0.002	0.056	4/12	FKBP5, TUBA1B, TUBB2B	27/72	1322
Formation of tubulin folding intermediates by CCT/TriC	4/30	0.002	0.068	2/2	TUBA1B, TUBB2B	17/30	250
Gap junction trafficking	5/52	0.002	0.073	3/20	TUBA1B, TUBB2B	18/52	1291
Separation of Sister Chromatids	10/194	0.002	0.073	5/8	ANAPC3, CDCA5, PPP2R1A, PSMB11, PSMD14, TUBA1B, TUBB2B	100/195	877
Cytokine signaling in Immune system	31/1055	0.003	0.087	23/639	EIF4G2, GBP6, HLA-C, HNRPDL, IL17, IRF5, KPNA3, MSN, NFKB2, PPP2R1A, PSMB11, PSMD14	479/1092	2005
Gap junction trafficking and regulation	5/56	0.003	0.089	3/24	TUBA1B, TUBB2B	19/56	1366
Class I MHC mediated antigen processing and presentation	17/464	0.003	0.092	26/48	ANAPC3, HLA-C, PSMB11, PSMD14	240/473	1377
Mitotic Anaphase	10/208	0.004	0.1	6/11	ANAPC3, CDCA5, PPP2R1A, PSMB11, PSMD14, TUBA1B, TUBB2B	124/249	1106
Cooperation of Prefolding and TriC/CCT in actin and tubulin folding	4/37	0.004	0.101	4/6	TUBA1B, TUBB2B	22/37	185
