# Supplementary material for: Functional Analysis of Autoantibody Signatures in Rheumatoid Arthritis
Source: Molecules. 2022 Feb 21;27(4):1452. doi: 10.3390/molecules27041452 (PMC8876797; doi:10.3390/molecules27041452)
Supplement: Supplementary file 1 [file molecules-27-01452-s001.zip › Supplementary Table S2.pdf]

Supplementary Table S2

Supplementary Table S2: Top 25 pathways enriched pathways for DIRAGs higher reactive in seronegative RA. Out of 221 cleaned IDs, 147 were found in Reactome pathway browser. Names (linked to Reactome pathway browser), found of total entities, their p-value and FDRs are given, the number of found out of total reactions, the found genesymbols within the pathway and results of the overrepresentation analysis for the complete chip are given (continued page 4-5).

Pathway name	found/total entities	entities p-value	entities FDR (BH)	found/total reactions	GeneSymbols found	found/total entities (16k)	rank (16k)
Antigen Presentation: Folding, assembly and peptide loading of class I MHC	36/102	1.11 * 10-16	1.31 * 10-14	14/16	HLA-A, HLA-C, SEC24C	84/102	2
Endosomal/Vacuolar pathway	35/82	1.11 * 10-16	1.31 * 10-14	3/4	HLA-A, HLA-C	74/82	1
ER-Phagosome pathway	37/164	1.11 * 10-16	1.31 * 10-14	4/10	HLA-A, HLA-C, PSMC3, PSMC5	122/173	3
Antigen processing-Cross presentation	37/186	1.11 * 10-16	1.31 * 10-14	8/23	HLA-A, HLA-C, PSMC3, PSMC5	127/195	22
Immunoregulatory interactions between a Lymphoid and a non-Lymphoid cell	38/316	1.11 * 10-16	1.31 * 10-14	12/43	C3, COL1A2, HLA-A, HLA-C, IGLC1	95/316	2008
Interferon gamma signaling	37/250	1.11 * 10-16	1.31 * 10-14	2/15	HLA-A, HLA-C	138/250	572
Interferon alpha/beta signaling	37/184	1.11 * 10-16	1.31 * 10-14	2/20	HLA-A, HLA-C	120/186	41
Interferon signaling	39/392	3.33 * 10-16	3.43 * 10-14	6/66	FLNA, HLA-A, HLA-C, NUP62	205/394	1102
Class I MHC mediated antigen processing and presentation	38/464	3.40 * 10-13	3.13 * 10-11	23/48	HLA-A, HLA-C, PSMC3, PSMC5, SEC24C	240/473	1377
Cytokine Signaling in Immune System	53/1,055	6.35 * 10-10	5.21 * 10-8	71/639	CCND1, COL1A2, FLNA, FN1, HLA-A, HLA-C, IL2RG, IL32, LCK, LCP1, NUP62, PSMC3, PSMC4, SHC1	479/1,092	2005

Adaptive Immune System	48/997	1.78 * 10-8	1.34 * 10-6	100/261	AP2A1, C3, CD74, COL1A2, CTSD, DNM2, DYNC1H1, HLA-A, HLA-C, IGLC1, LCK, MTOR, PSMC3, PSMC5, SEC24C	393/1,004	2031
Transcriptional regulation by RUNX3	10/118	1.67 * 10-4	0.012	15/47	CCND1, CTGF, JAG1, KAT2A, NOTCH1, PSMC3, PSMC5	61/118	646
Immune system	77/2,641	3.16 * 10-4	0.02	304/1,493	ACTB, AP2A1, APEH, APRT, C3, CASP8, CCND1, CCT8, CD74, COL1A2, CTSD, DDOST, DNM2, DSP, DYNC1H1, EEF1A1, FLNA, FN1, GUSB, HLA-A, HLA-C, IGLC1, IL2RG, IL32, IMPDH2, LCK, LCP1, MTOR, MUC5B, MYO1C, NUP62, PSMC3, PSMC5, RNF216, SEC42C, SHC1, VCL	962/2,681	2039
RUNX3 regulates NOTCH signaling	4/16	3.39 * 10-4	0.02	7/7	JAG1, KAT2A, NOTCH1	9/16	234
Interleukin-2 signaling	3/14	0.003	0.164	11/18	IL2RG, LCK, SHC1	7/14	374
SHC-related events triggered by IGF1R	3/15	0.004	0.184	5/5	SHC1	4/16	1,078
MET activates PTK2 signaling	4/32	0.004	0.203	1/5	COL1A2, FN1, ITGA3, LAMC1	16/32	451
Extracellular matrix organization	14/329	0.007	0.341	103/318	ADAM19, ADAMTS4, COL1A2, COMP, CTSD, FN1, GLT25D1, HSPG2, ITGA3, LAMC1, LTBP3, PLOD1, SERPINH1	118/329	1,952
Nef-mediates down modulation of cell surface receptors by recruiting them to clathrin adapters	3/22	0.01	0.419	11/15	AP2A1, HLA-A, LCK	14/22	122

Constitutive Signaling by NOTCH1 t(7;9) Translocation Mutant	2/8	0.012	0.419	5/5	JAG1, NOTCH1	2/8	846
Signaling by NOTCH1 t(7;9) Translocation Mutant	2/8	0.012	0.419	5/5	JAG1, NOTCH1	2/8	845
RHO GTPases activate CIT	3/23	0.012	0.419	6/6	CIT, MYH14	13/23	244
MET promotes cell motility	4/45	0.014	0.419	1/12	COL1A2, FN1, ITGA3, LAMC1	21/45	647
RUNX3 regulates YAP1-mediated transcription	2/9	0.014	0.419	2/3	CTGF	4/9	414
RUNX1 regulation transcription of genes involved in WNT signaling	2/9	0.014	0.419	2/4	AXIN1	3/9	648
