# Supplementary material for: Functional Analysis of Autoantibody Signatures in Rheumatoid Arthritis
Source: Molecules. 2022 Feb 21;27(4):1452. doi: 10.3390/molecules27041452 (PMC8876797; doi:10.3390/molecules27041452)
Supplement: Supplementary file 1 [file molecules-27-01452-s001.zip › Supplementary Table S3.pdf]

Supplementary Table S3

Supplementary Table S3: Top 25 enriched pathways for DIRAGs higher reactive in established PIA. Out of the 988 cleaned IDs, 673 were found in Reactome pathway browser. Names (linked to Reactome pathway browser), found of total entities, their p-value and FDRs are given, the number of found out of total reactions, the found genesymbols within the pathway and results of the overrepresentation analysis for the complete chip are given (continued page 7-10).

Pathway name	found/total entities	entities p-value	entities FDR (BH)	found/total reactions	GeneSymbols found	found/total entities (16k)	rank (16k)
Endosomal/vacuolar pathway	36/82	$1.33 * 10^{-15}$	$2.08 * 10^{-12}$	3/4	HLA-A, HLA-C, HLA-E	74/82	1
Antigen Presentation: Folding, assembly and peptide loading of class I MHC	37/102	$1.68 * 10^{-13}$	$1.31 * 10^{-10}$	13/16	HLA-A, HLA-C, HLA-E, SEC13	84/102	2
Interferon alpha/beta signaling	50/184	$5.11 * 10^{-13}$	$2.66 * 10^{-10}$	14/20	ADAR, HLA-C, HLA-E, IFITM1, IRF4, ISG15, PSMB8, TYK2	120/186	41
ER-Phagosome pathway	46/165	$1.86 * 10^{-12}$	$7.29 * 10^{-10}$	5/10	HLA-A, HLA-C, HLA-E, MAL, PSMA1, PSMA2, PSMB1, PSMB8, PSMC2, PSMC4, PSMD10, PSMD13, UBC	122/173	3
Interferon Signaling	77/392	$4.17 * 10^{-12}$	$1.31 * 10^{-9}$	33/66	ADAR, EIF2AK2, EIF4E, EIF4G1, EIF4G2, FLNA2, FLNB, GBP5, GBP6, HLA-A, HLA-C, HLA-DPB1, HLA-E, IFITM1, IRF4, ISG15, KPNA2, MAPK3, NUP107, NUP210, NUP62, PSMB8, SEC13, SP140L, STAT4, TRIM22, TRIM8, TYK2, UBA7, UBC, UBE2N	205/394	1102
Antigen processing-Cross presentation	47/187	$8.84 * 10^{-9}$	$8.84 * 10^{-9}$	10/23	HLA-A, HLA-C, HLA-E, MAL, NCF2, PSMA1, PSMA2, PSMB1, PSMB8, PSMC2, PSMC4, PSMD10, PSMD13, UBC	127/195	22
Interferon gamma signaling	52/250	$4.69 * 10^{-7}$	$4.69 * 10^{-7}$	2/15	GBP5, GBP6, HLA-A, HLA-C, HLA-DPB1, HLA-E, IRF4, SP140L, TRIM22, TRIM8	138/250	572

Cytokine Signaling in Immune System	160/1,245	1.73 * 10^{-6}	1.73 * 10^{-6}	402/699	ADAR, ARAF, ARF5, ARRB2, ATF1, BCL6, BTRC, CCND1, CDKN1A, COLIA2, CSF1R, CUL1, CUL3, EIF2AK2, EIF4E, EIF4G1, EIF4G2, ERBB3, FAN1, FGFR1, FGFR3, FLNA, FLNB, FN1, GBP5, GBP6, GRIN1, HIF1A, HLA-A, HLA-C, HLA-DPB1, HLA-E, HNRPDL, HSP90AA1, HSP90B1, IFITM1, IL10RA, IL16, IL2RB, IQGAP1, IRAK1, IRF4, ISG15, KPNA2, LAT, LCP1, LGAL59, MAP3K11, MAP3K14, MAPK2, MSN, MUP107, NUP210, NUP62, PEBP1, PIK3CD, PIM1, PRKACA, PSMA1, PSMA2, PSMB1, PSMB8, PSMC2, PSMC4, PSMD10, PSMD13, PTK2B, PTPN7, RAPGEF1, RASA3, RASAL13, S100A9, SEC13, SMARCA4, SP140L, SPTAN1, SPTBN1, STAT3, STAT4, STAT5A, STAT6, STX1A, TALDO1, TNFRSF1B, TNFRSF4, TRIM22, TRIM8, TYK2, UBA7, UBC, UBE2M, UBE2N, VAV1, VCL, ZEB1	479/1092	2005
GTP hydrolysis and joining of the 60S ribosomal subunit	31/120	6.25 * 10^{-6}	6.25 * 10^{-6}	3/3	EIF3H1, EIF3M, EIF4B, EIF4E, EIF4G1, EIF4H, RPL10A, RPL12, RPL15, RPL17, RPL22, RPL24, RPL26, RPL27, RPL27A, RPL4, RPL41, RPL7, RPS10, RPS18, RPS19, RPS25, RPS4Y2, RPS5, RRP1, TGM2	75/120	96
L13a-mediated translational silencing of Ceruloplasmin expression	31/120	6.25 * 10^{-6}	6.25 * 10^{-6}	2/3	EIF3A, EIF3B, EIF3G, EIF3H, EIF3M, EIF4B, EIF4E, EIF4G1, EIF4H, RPL10A, RPL12, RPL15, RPL17, RPL22, RPL24, RPL26, RPL27, RPL27A, RPL4, RPL41, RPL7, RPS10, RPS18, RPS19, RPS25, RPS4Y2, RPS5, RRP1, TGM2	75/120	95
Cap-dependet Translation Initiation	32/130	9.29 * 10^{-6}	9.29 * 10^{-6}	16/18	EIF2B4, EIF3A, EIF3B, EIF3G, EIF3H, EIF3M, EIF4B, EIF4E, EIF4G1, EIF4H, RPL10A, RPL12, RPL15, RPL17, RPL22,	79/130	147

					RPL24, RPL26, RPL27, RPL27A, RPL4, RPL41, RPL7, RPS10, RPS18, RPS19, RPS25, RPS4Y2, RPS5, RRP1, TGM2		
Eukaryotic Translation Initiation	32/130	$9.29 * 10^{-6}$	$9.29 * 10^{-6}$	18/21	EIF2B4, EIF3A, EIF3B, EIF3G, EIF3H, EIF3M, EIF4B, EIF4E, EIF4G1, EIF4H, RPL10A, RPL12, RPL15, RPL17, RPL22, RPL24, RPL26, RPL27, RPL27A, RPL4, RPL41, RPL7, RPS10, RPS18, RPS19, RPS25, RPS4Y2, RPS5, RRP1, TGM2	79/130	146
Class I MHC mediated antigen processing & presentation	72/465	$3.04 * 10^{-7}$	$3.66 * 10^{-5}$	30/48	BIRC6, BTRC, CUL1, CUL3, FBXL19, FBXO21, FBXW5, HLA-A, HLA-C, HLA-E, KCTD7, KEAP1, MAL, NARF, NCF2, PSMA1, PSMA2, PSMB1, PSMB8, PSMC2, PSMC4, PSMD10, PSMD13, RNF19A, RNF213, SEC13, SIAH2, STUB1, TRIM41, UBA1, UBA7, UBC, UBE2J2, UBE2M, UBE2N, UBR4, WWP1, ZNRF1	240/473	1377
Formation of a pool of free 40S subunits	27/106	$3.84 * 10^{-7}$	$4.26 * 10^{-5}$	2/2	EIF3A, EIF3B, EIF3G, EIF3H, EIF3M, RPL10A, RPL12, RPL15, RPL17, RPL22, RPL24, RPL26, RPL27, RPL27A, RPL4, RPL41, RPL7, RPS10, RPS18, RPS19, RPS25, RPS4Y2, RPS5, RRP1, TGM2	66/106	101
Major pathway of rRNA processing in the nucleolus and cytosol	38/189	$6.77 * 10^{-7}$	$7.04 * 10^{-5}$	7/7	BYSL, CSNK1E, DDX21, EXOSC10, EXOSC7, GNL3, IMP4, LTV1, NCL, NOC4L, NOL11, PDCD11, RIOK3, RPL10A, RPL12, RPL15, RPL17, RPL22, RPL24, RPL26, RPL27, RPL27A, RPL4, RPL41, RPL7, RPS10, RPS18, RPS19, RPS25, RPS4Y2, RPS5, RRP1, SKIV2L2, UTP6, WDR18	109/189	334

Metabolism of RNA	102/782	3.01 * 10^{-6}	2.92 * 10^{-4}	92/187	ADAR, BYSL, C22orf28, C2orf9, CNOT1, CSNK1E, CSTF2T, DDX1, DDX21, DDX39A, EDC4, EFTUD2, EIF4B, EIF4E, EIF4C1, ELAVL1, ERCC3, EXOSC10, EXOSC7, GNL3, FTF2F1, HNRNPA0, HNRNPC, HNRNPD, HNRNPM, HNRNPR, IMP4, LTV1, NCL, NOC4L, NOL11, NT5C3L, NUP107, NUP210, NUP62, PDCD11, POLR2L, PRKACA, PRKCA, PRPF31, PRPF6, PSMA1, PSMA2, PSMB1, PSMB8, PSMC2, PSMC4, PSMD10, PSMD13, PTBP1, PUF60, PUS3, RIOK3, RPL10A, RPL12, RPL15, RPL17, RPL22, RPL24, RPL26, RPL27, RPL27A, RPL4, RPL41, RPL7, RPS10, RPS18, RPS19, RPS25, RPS4Y2, RPS6, RRP1, SEC13, SF1, SF3B4, SKIV2L, SKIV2L2, SLU7, SMG5, SMG7, SMG8, SRRM2, SRSF2, SRSF3, SRSF5, SRSF7, SUPT5H, THOC2, TNKS1BP1, TSEN54, U2AF2, UBC, UPF1, UTP6, WDR19, XPOT	387/782	1712
rRNA processing in the nucleus and cytosol	38/207	5.42 * 10^{-6}	4.98 * 10^{-4}	8/15	BYSL, CSNK1E, DDX21, EXOSC10, EXOSC7, GNL3, IMP4, LTV1, NCL, NOC4L, NOL11, PDCD11, RIOK3, RPL10A, RPL12, RPL15, RPL17, RPL22, RPL24, RPL26, RPL27, RPL27A, RPL4, RPL41, RPL7, RPS10 RPS18, RPS19, RPS25, RPS4Y2, RPS5, RRP1, SKIV2L2, UPT6, WDR18	113/207	579
Eukaryotic Translation Elongation	24/102	6.20 * 10^{-6}	5.33 * 10^{-4}	8/9	EEF1A1, EEF1D, EEF1G, RPL10A, RPL12, RPL15, RPL17, RPL22, RPL24, RPL26, RPL27, RPL27A, RPL4, RPL41, RPL7, RPS10, RPS18, RPS19, RPS25, RPS4Y2, RPS5, RRP1	60/102	212
Nonsense Mediated Decay (NMD) independet of the	23/101	1.59 * 10^{-5}	0.001	1/1	EIF4G1, RPL10A, RPL12, RPL15, RPL17, RPL22, RPL24, RPL26, RPL27, RPL27A,	62/101	126

Exon Junction Complex (EJC)					RPL4, RPL41, RPL7, RPS10, RPS18, RPS19, RPS25, RPS4Y2, RPS5, RRP1, UPF1		
Translation	52/339	1.71 * 10^{-5}	0.001	65/99	AARS2, APEH, AURKA1P1, CCDC142, DDOST, EEF1A1, EEF1D, EEF1G, EIF2B4, EIF3A, EIF3B, EIF3G, EIF3H, EIF3M, EIF4B, EIF4E, EIF4G1, EIF4H, FARSA, HARS, LARS, MRPL4, MRPL54, MRPS6, OXA1L, PARS2, PPA1, RPL10A, RPL12, RPL15, RPL17, RPL22, RPL24, RPL26, RPL27, RPL27A, RPL4, RPL41, RPL7, RPS10, RPS18, RPS19, RPS25, RPS4Y2, RPS5, RRP1, SARS, TGM2, VARS, YARS	149/339	1710
Nonsense-Mediated Decay (NMD)	26/124	1.86 * 10^{-5}	0.001	6/6	EIF4G1, RPL10A, RPL12, RPL15, RPL17, RPL22, RPL24, RPL26, RPL27, RPL27A, RPL4, RPL41, RPL7, RPS10, RPS18, RPS19, RPS25, RPS4Y2, RPS5, RRP1, UPF1	72/124	273
Nonsense Mediated Decay (NMD) enhanced by the Exon Junction Complex (EJC)	26/124	1.86 * 10^{-5}	0.001	5/5	EIF4G1, RPL10A, RPL12, RPL15, RPL17, RPL22, RPL24, RPL26, RPL27, RPL27A, RPL4, RPL41, RPL7, RPS10, RPS18, RPS19, RPS25, RPS4Y2, RPS5, RRP1, SMG5, SMG7, SMG8, UPF1	72/124	274
Translation initiation complex formation	17/62	2.11 * 10^{-5}	0.001	2/2	EIF3A, EIF3B, EIF3G, EIF3H, EIF3M, EIF4B, EIF4E, EIF4G1, EIF4H, RPS10, RPS18, RPS19, RPS25, RPS4Y2, RPS5, TGM2	37/62	184
Peptide chain elongation	22/97	2.55 * 10^{-5}	0.002	4/5	EEF1A1, RPL10A, RPL12, RPL15, RPL17, RPL22, RPL24, RPL26, RPL27, RPL27A, RPL4, RPL41, RPL7, RPS10, RPS18, RPS19, RPS25, RPS4Y2, RPS5, RRP1	57/97	213
Ribosomal scanning and start codon recognition	17/64	3.12 * 10^{-5}	0.002	2/2	EIF3A, EIF3B, EIF3G, EIF3H, EIF3M, EIF4B, EIF4E, EIF4G1, EIF4H, RPS10, RPS18, RPS19, RPS25, RPS4Y2, RPS5, TGM2	36/64	296
