# Supplementary material for: Functional Analysis of Autoantibody Signatures in Rheumatoid Arthritis
Source: Molecules. 2022 Feb 21;27(4):1452. doi: 10.3390/molecules27041452 (PMC8876797; doi:10.3390/molecules27041452)
Supplement: Supplementary file 1 [file molecules-27-01452-s001.zip › Supplementary Table S4.pdf]

Supplementary Table S4

***Supplementary Table S4:** The enriched pathways for antigens **positively (A)** and the **top 20 negatively (B)** correlated with CDAI. Out of the 429 antigenic proteins found significantly correlated with CDAI of both seropositive and seronegative RA patients, 153 positively correlated and 276 negatively correlated proteins were subjected separately to Reactome pathway analysis. Names (linked to Reactome pathway browser), found and total entities, ratio, their p-value and FDRs are given, the number of found & total reactions, as well the respective ratio are given.*

A) POSITIVELY CORRELATED Pathway name	Entities found	Entities Total	Entities ratio	Entities pValue	Entities FDR	Reactions found	Reactions total	Reactions ratio
RNA Polymerase I Transcription Initiation	4	50	0.003	2.86E-03	3.37E-01	*4*	*6*	*0*
RUNX1 regulates expression of components of tight junctions	2	8	0.001	3.99E-03	3.37E-01	*2*	*6*	*0*
Metabolism of RNA	18	785	0.052	4.49E-03	3.37E-01	*70*	*187*	*0.014*
Epigenetic regulation of gene expression	6	139	0.009	5.85E-03	3.37E-01	*6*	*34*	*0.002*
Cyclin A/B1/B2 associated events during G2/M transition	3	32	0.002	6.31E-03	3.37E-01	*3*	*25*	*0.002*
RNA Polymerase I Transcription Termination	3	33	0.002	6.86E-03	3.37E-01	*3*	*4*	*0*
Nef mediated downregulation of MHC class I complex cell surface expression	2	11	0.001	7.38E-03	3.37E-01	*3*	*4*	*0*
Formation of the HIV-1 Early Elongation Complex	3	34	0.002	7.44E-03	3.37E-01	*5*	*5*	*0*
Formation of the Early Elongation Complex	3	34	0.002	7.44E-03	3.37E-01	*3*	*3*	*0*
mRNA Capping	3	35	0.002	8.05E-03	3.37E-01	*11*	*11*	*0.001*
Glycolysis	5	110	0.007	9.42E-03	3.37E-01	*3*	*24*	*0.002*
Activation of PPARGC1A (PGC-1alpha) by phosphorylation	2	13	0.001	1.02E-02	3.37E-01	2	2	0

B) NEGATIVELY CORRELATED<br>Pathway name	Entities found	Entities Total	Entities ratio	Entities pValue	Entities FDR	Reactions found	Reactions total	Reactions ratio
Antigen Presentation: Folding, assembly and peptide loading of class I MHC	37	103	0.007	1.11E-16	1.57E-14	*13*	*16*	*0.001*
Endosomal/Vacuolar pathway	36	82	0.005	1.11E-16	1.57E-14	*3*	*4*	*0*
Class I MHC mediated antigen processing & presentation	51	474	0.031	1.11E-16	1.57E-14	*28*	*48*	*0.004*
ER-Phagosome pathway	44	173	0.011	1.11E-16	1.57E-14	*4*	*10*	*0.001*
Interferon Signaling	50	394	0.026	1.11E-16	1.57E-14	*25*	*71*	*0.005*
Antigen processing-Cross presentation	44	195	0.013	1.11E-16	1.57E-14	*8*	*23*	*0.002*
Interferon alpha/beta signaling	45	188	0.012	1.11E-16	1.57E-14	*6*	*24*	*0.002*
Interferon gamma signaling	43	250	0.017	1.11E-16	1.57E-14	*2*	*16*	*0.001*
Immunoregulatory interactions between a Lymphoid and a non-Lymphoid cell	37	316	0.021	2.11E-15	2.64E-13	*10*	*44*	*0.003*
Cytokine Signaling in Immune system	70	1,092	0.072	1.37E-14	1.54E-12	*67*	*710*	*0.052*
Adaptive Immune System	59	1,005	0.067	7.67E-11	7.83E-09	*82*	*264*	*0.019*
Immune System	99	2,684	0.178	1.26E-06	1.19E-04	*238*	*1,625*	*0.119*
AUF1 (hnRNP D0) binds and destabilizes mRNA	8	56	0.004	6.34E-05	5.51E-03	*3*	*4*	*0*
Regulation of mRNA stability by proteins that bind AU-rich elements	10	93	0.006	8.41E-05	6.73E-03	*12*	*26*	*0.002*
ROS sensing by NFE2L2	8	60	0.004	1.02E-04	7.62E-03	*3*	*8*	*0.001*

HIV Infection	17	263	0.017	1.94E-04	1.26E-02	*35*	*160*	*0.012*
Regulation of activated PAK-2p34 by proteasome mediated degradation	7	50	0.003	2.05E-04	1.26E-02	*2*	*2*	*0*
APC/C:Cdc20 mediated degradation of Securin	8	68	0.005	2.36E-04	1.26E-02	*3*	*3*	*0*
Vpu mediated degradation of CD4	7	53	0.004	2.90E-04	1.26E-02	*2*	*5*	*0*
p53-Independent G1/S DNA damage checkpoint	7	54	0.004	3.24E-04	1.26E-02	*2*	*4*	*0*
